# Neurotic personality trait as a predictor in the prognosis of composite restorations: A 24-month clinical follow up study

**DOI:** 10.1038/s41598-021-96229-3

**Published:** 2021-08-25

**Authors:** Sulthan Ibrahim Raja Khan, Dinesh Rao, Anupama Ramachandran, Bhaskaran Veni Ashok, Jagan Kumar Baskaradoss

**Affiliations:** 1 Department of Conservative Dentistry & Endodontics, Pacific Dental College, Pacific Academy of Higher Education and Research University, Pacific Hills, Pratap Nagar Extension, Airport Road, Udaipur, 313003 Rajasthan India; 2 Department of Pedodontics & Preventive Dentistry, Pacific Dental College, Pacific Academy of Higher Education and Research University, Udaipur, Rajasthan India; 3 Department of Conservative Dentistry & Endodontics, Chettinad Dental College, Kelambakkam, Tamil Nadu India; 4 Department of Conservative Dentistry & Endodontics, Ragas Dental College, Uthandi, Tamil Nadu India; 5 Department of Developmental and Preventive Sciences, Faculty of Dentistry, Kuwait University, Jabriya, Kuwait

**Keywords:** Diseases, Health care, Risk factors

## Abstract

The role of personality traits in modulating the incidence and progression of medical disease conditions are well documented, however, there is a paucity of information for its effects on dental health conditions and specifically on the prognosis of restorative dental materials. This study aims to evaluate the clinical performance of Micro-hybrid and Nano-ceramic composite restorations among patients with different personality traits. A total of 323 patients, indicated to receive operative treatment at a University Dental College Hospital, were invited to participate in this study. Consenting patients were requested to complete the Big Five Inventory (BFI-44 Item) personality questionnaire and were evaluated by a psychiatrist for categorizing the participants based on their personality traits. Out of the recruited patients, 124 patients falling in to the dominant trait of Agreeableness (n = 62) and Neuroticism (n = 62) were included in the study for further investigation. Next, patients from the Agreeableness (Group A) and the Neuroticism personality trait group (Group N) were randomly divided into two subgroups each—sub group Am (n = 44) and Nm (n = 48) for Micro-hybrid composite restorations and Sub group An (n = 42) and Nn (n = 47) for Nano-ceramic composite restorations. Two trained and calibrated dentists prepared the cavities according to previously published methodology. The restorations were evaluated at baseline (immediately after restoration), 6-months, 12-months and 24-months intervals by two blinded independent dental professionals for anatomical form, secondary caries, color match, retention, marginal adaptation, surface texture, marginal discoloration and post-operative sensitivity. There is no statistically significant difference noted in various parameters of restoration performance between Micro-hybrid composite and Nano-ceramic composite compared among ‘agreeableness’ personality group and among ‘neuroticism’ personality group after controlling the personality trait factor. Higher ‘Neuroticism’ individuals had higher restoration deterioration in color matching and surface texture when compared to higher ‘Agreeableness’ trait individuals. Regression analysis showed no effect of gender or cavity size on the outcome of results. Assessment of personality traits may serve as a useful tool during treatment planning which would aid clinicians in choosing suitable restorative dental material and prosthesis design according to individual patient’s physiological and functional needs, thereby overall improving the quality of treatment provided.

## Introduction

Personality can be defined as the "dynamic organization of the psychobiological systems that modulate adaptation to changing environments through several personality traits, which are long-lasting patterns of how we perceive, relate to, and think about oneself, other people and the world as a whole"^1^. Among various models of personality assessment, Costa and McCrae’s five-factor model is one of the widely used model in personality evaluation, it divides personality traits in to five dimensions which include neuroticism, extraversion, openness, agreeableness, and conscientiousness^2,3^.

Individuals with higher levels of neurotic traits experience higher amount of stress, anxiety, irritability, low self-esteem, poor emotional control, minor depressions, excessive smoking and alcohol addiction. They are unable to control cravings and urges^4^. Whereas, higher agreeableness individuals are warm, cooperative, kind, considerate, trustworthy and selfless natured thus maintaining positive interpersonal relationship with others^5^.

Higher levels of neuroticism trait has numerous health implications^6^. Malouff et al.^7^ in their meta-analysis examined the relationship between the personality traits and clinical disorders and observed that higher neuroticism had a strong correlation with the incidence of mental and physical health problems than other personality traits. However people with higher agreeableness are better in self-regulating their behavior and experience better subjective mental and physical health^8^.

From a dental viewpoint, there is strong association between the characteristics of bruxist and higher neuroticism trait individuals^9^. Bruxism is proved to be a risk factor in the survival of prosthetic and implant restorations^10^. Thus, the current study examines the significance of higher neuroticism trait’s impact on restorative treatment procedures.

Physical characteristics of composite materials are supremely important in restorative dentistry^11,12^. However still there is confusion regarding choice of suitable restorative materials. Recently, nanotechnology has been introduced in the dentistry that contains small particles of nanometers ranges. These small particles are favorable to obtain good wear resistance, polishability and better esthetics^13^. Many researchers observed that Nano-hybrid composites performed better in all mechanical properties tested than Micro-hybrid composites^14,15^. However, few other systematic reviews found no difference between Micro-hybrid and Nano-hybrid composite^16,17^, and no clear consensus has still been achieved.

There are many studies done in investigating the role of personality traits in modulating the incidence and progression of medical disease conditions; however, there are hardly few studies for the same in dental health conditions and a literature search revealed that there are no studies for the same in restorative dentistry.

Hence, the main objective of the study is to clinically evaluate the performance of restorations using United States Public Health Service (USPHS) clinical evaluation criteria in patients with different personality trait. Also, the current study aims to observe the influence of newer materials like Nano-ceramic on the outcome of the restoration over a period of 24-months after controlling and standardization of various factors, which can affect the restoration’s prognosis.

## Material and methods

All subjects gave their informed consent for participation in this study. The study was conducted in accordance with the Declaration of Helsinki, and the protocol was approved by the Ethics Committee of the institute where the study was carried out. The inclusion criteria were age between 20–35 years and presence of full set of permanent dentition (at least 28 excluding third molars) with a bilateral angle class I molar and canine relationship. Patients having large occlusal restorations, presence of endodontically treated teeth, fixed prostheses or active periodontal disease was excluded. Also, presence of local or systemic osseous or neuromuscular disease, presence of spontaneous orofacial pain, temporomandibular joint disorders, large facial asymmetry and pregnancy were not considered for the study.

### Personality evaluation

A total of 323 patients, in accordance with the inclusion and exclusion criteria were given the Big Five Inventory-44 item^18,19^ to fill and were evaluated accordingly with the help of a psychiatrist. The patients were subsequently allotted to the various personality trait group as per their dominant trait score (Appendix 1). The inventory has 44 items that are rated in a 5 point Likert scale ranging from 1 (strongly disagree) to 5 (strongly agree). BFI Inventory is a robust personality assessment tool which is widely used among diverse countries, cultures and languages including several samples in India. The data suggest that Indian samples displayed similarities in personality structure to more than 50 other cultural or linguistic groups especially in traits of Neuroticism, Agreeableness and Conscientiousness with slight to moderate variation in Extraversion and Openness^20,21^. BFI personality constructs are biologically based and that “cultures shape the expression of traits”^22^. BFI-44 item inventory is found to be reliable when studied among the same city population before^23^.

### Analysis of BFI score

BFI scores were calculated using a continuous weighted dimension score method (sum of items score divided by the number of items completed)^24^. Among patients who had agreeableness and neuroticism as their dominant trait, only those who had a cut off score ≥ 27 and ≥ 29 respectively were selected for further study investigation (Table 1). As there is an insufficient normative mean scores or specific cut off scores for this studied population, third quartile scores were used as cut off scores to ensure the presence of strong dominant trait. Of the studied sample, 62 had strong agreeableness trait score and 81 patients had strong neuroticism trait score.

**Table 1:** Descriptive statistics for BFI.

N(323)	Minimum score	Maximum score	Mean score	Std deviation	1st quartile	3rd quartile
Extraversion	15	40	21.31	5.64	17	24
Agreeableness	11	39	21.45	7.05	16	27
Conscientiousness	9	45	19.97	7.04	15	23
Neuroticism	8	39	20.72	8.43	14	29
Openness	10	48	26.50	8.91	20	33.5

### Dental procedural part

124 patients falling into the Agreeableness (n = 62) and Neuroticism (n = 62) traits were included in the study for cavity preparation, restoration and USPHS evaluations.

### Randomization

The selected 62 patients of each dominant personality group were numbered. They were further divided in to odd and even numbered sub groups. The choice of micro-hybrid and nano-ceramic restoration between the odd and even sub groups was decided by tossing a coin. Hence, patients from the Agreeableness (Group A) and the Neuroticism (Group N) were divided (Irrespective of gender difference) into two subgroups each—Sub Group Am (n = 44; 31small size, 13 moderate size) and Sub Group Nm (n = 48; 31small size,17 moderate size) for micro-hybrid composite restorations and Sub Group An (n = 42; 31small size,11 moderate size) and Sub Group Nn (n = 47; 32 small size,15 moderate size) for Nano-ceramic composite restorations.

*Group Nomenclature: Agreeableness(A), Neuroticism(N), Micro-hybrid(m), Nano-ceramic(n).

### Clinical procedure

Two clinical dentists performed the operative procedure. Class I cavities of Small size (cavity extended less than 1/4th of the way up the cuspal slopes) and moderate size (cavity extended between ¼ and 1/3rd of the way up the cuspal slopes) were prepared in molar teeth according to previous methodology^25^. The outlines of the preparations were limited to the removal of caries/defective restoration. The teeth were etched (DeTrey Conditioner 36, Dentsply Sirona, Konstanz, Germany), self-priming adhesive resin bonding agent (Prime & Bond NT, Dentsply Sirona, Konstanz, Germany) was applied and cured as per manufacturer instructions. Sixty seven patients received one restoration and fifty seven patients received two restorations but of the same generic category either the micro-hybrid (Spectrum TPH, Dentsply/De trey, Germany) or the Nano-ceramic composite (Ceram X mono, Dentsply/De trey, Germany) [Table 2]. Placement of resin composites were done employing incremental technique and were cured with the LED light (minimum irradiance output between 550 mW/cm^2^ and 800 mW/cm^2^) for 40 s by uniform continuous curing technique followed by finishing and polishing procedures (Enhance System Kit, Dentsply Sirona, Germany).

**Table 2:** Material Composition and Batch Number.

Material name	Material type	Filler volume/weight	Composition	Manufacturer & batch number
SPECTRUM T.P.H	Microhybrid	57 vol % / 77 wt %	Matrix: Bis-GMA, Bis-EMA, TEGDMA Barium-aluminium -borosilicate Glass (mean particle size < 1 μm), Highly dispersed silicon dioxide (particle size 0.04 μm)	Dentsply De Trey GmbH, Konstanz, Germany 60,605,301 60,605,302 60,605,303
CERAM.X MONO	Nanoceramic	57 vol % / 76 wt %	Methacrylate modified polysiloxane, dimethacrylate Barium-aluminum borosilicate Glass (mean particle size 1.2–1.6 μm), methacrylate functionalized silicon dioxide (nano filler, 10 nm)	Dentsply De Trey GmbH, Konstanz, Germany 60,701,511 60,701,512 60,701,513

### Evaluation

At the baseline (immediately after restoration), 6-months, 12-months and 24-months; the restorations were evaluated by two double blinded (had no knowledge about both personality and restoration type) independent dental professionals for anatomical form, secondary caries, color match, retention, marginal adaptation, surface texture, marginal discoloration, post-operative sensitivity using the Modified USPHS evaluation criteria^26–28^ and any variation over evaluations were solved through discussion to reach consensus by both examiners (Appendix II). The study's Principal Investigator conducted calibration of the evaluators prior to the study evaluation. Excellent inter-examiner reliability (kappa > 0.80) for USPHS scorings were observed, as assessed in a subsample of 20 patients not included in the study.

### Statistical analysis

As the evaluation of restorations provided only ordinal structural data, non-parametric statistical analysis (Mann–Whitney U-test) was performed using an SPSS software program (SPSS version 20.0, Chicago, IL, USA)and a probability value of (P < 0.05) was considered to be statistically significant. Mean Rank Scores were compared among groups at different time points and lower mean rank score denotes better restoration performance and Vice-Versa. Regression analysis was performed to observe the effect of gender and cavity size on study results.

### Ethics approval and consent to participate

Guidelines documented in the Helsinki-2013 Declaration of experiments on humans were adopted for this study. The ethics committee of the Ragas Dental College Hospital, Tamil Nadu Dr. M.G.R Medical University, approved the study protocol (ethical clearance number 20180730). Participating individuals were mandated to read and signed a consent form. Prior to signing the consent form, all participating patients were informed that they could withdraw from this study at any stage without any penalty; and were invited to ask questions.

## Results

Total of nine patients were lost to follow up (Table 3). At 24 month evaluation, majority of the restorations scored A(88.7%), few scored B and C(10.7%) among all groups, however total restoration failure (Requiring replacement) is observed only in neuroticism group which is 2.17% (Group Nm) and 6.81% (Group Nn). There is no statistically significant difference noted in various parameters of restoration performance between Micro-hybrid composite (Sub Group m) and Nano-ceramic composite (Sub Group n) compared among Agreeableness personality (Group A) population and among Neuroticism personality (Group N) population after controlling the personality trait factor (Tables 4 and 5). There is no statistically significant difference noted in various parameters of Micro-hybrid composite restoration performance compared between Agreeableness personality (Group Am) and Neuroticism personality (Group Nm) population (Table 6). However, there is statistically significant difference noted in 24-month evaluation only on color matching and surface texture parameter of Nano-ceramic composite restoration compared between Agreeableness personality (Group An) and Neuroticism personality (Group Nn) groups (Table 7). Neuroticism individuals had increased restoration deterioration in color matching and surface texture when compared to Agreeableness trait individuals. Also, there is no statistically significant difference noted in various parameters of combined (Micro-hybrid plus Nano-ceramic) restoration’s performance between Agreeableness personality (Group A) population and Neuroticism personality (Group N) population (Table 8). Inter Rater-Reliability (kappa) score between both evaluators had almost perfect agreement between them except for secondary caries in which they had substantial agreement (Table 9). Regression analysis showed no effect of gender or cavity size on the outcome of results (Supplementary Tables 10–25).

**Table 3:** Descriptive statistics at 24- month recall.

Evaluation criteria	Score	Group A Total		Group Am	Group An	Group N Total		Group Nm	Group Nn
		N(86)	%	N(44)	N(42)	N(95)	%	N(48)	N(47)
Losses to follow up		4	95.3	3	1	5	94.7	2	3
Retention	A	82	100	41	41	88	97.7	46	42
	B	0	0	0	0	2	2.2	0	2
Color Match	A	72	87.8	36	36	70	77.7	38	32
	B	10	12.1	5	5	18	20	8	10
	C	0	0	0	0	2	2.2	0	2
Marginal Discoloration	A	69	84.1	34	35	70	77.7	37	33
	B	13	15.8	7	6	19	21.1	9	10
	C	0	0	0	0	1	1.1	0	1
Marginal Adaptation	A	75	91.4	38	37	75	83.3	41	34
	B	7	8.5	3	4	15	16.6	5	10
Secondary Caries	A	76	92.6	38	38	84	93.3	44	40
	B	6	7.3	3	3	6	6.6	2	4
Surface texture	A	70	85.3	35	35	66	73.3	36	30
	B	12	14.6	6	6	23	25.5	10	13
	C	0	0	0	0	1	1.1	0	1
Anatomic Form	A	78	95.1	39	39	84	93.3	44	40
	B	4	4.8	2	2	6	6.6	2	4
Postoperative Sensitivity	A	78	95.1	40	38	84	93.3	44	40
	B	4	4.8	1	3	6	6.6	2	4

*Scores A, B and C are qualitative variables. A denote success, Band C denote failure in increasing order.

*The groups are coded as follows:

*Group Am* AGREEABLENESS (micro-hybrid), *Group An* AGREEABLENESS (nano-ceramic), *Group Nm* NEUROTICISM (micro-hybrid), *Group Nn* NEUROTICISM (nano-ceramic).

**Table 4:** Comparison of Group Am with Group An at baseline, 6, 12 and 24 months among different parameters.

		Groups	N	Mean rank	P value
Retention	Baseline	Group Am	44	43.5	1
		Group An	42	43.5	
	6 Months	Group Am	44	43.98	0.329
		Group An	42	43	
	12 Months	Group Am	44	43.98	0.329
		Group An	42	43	
	24 Months	Group Am	44	44.43	0.332
		Group An	42	42.52	
Color match	Baseline	Group Am	44	43.48	0.974
		Group An	42	43.52	
	6 Months	Group Am	44	43.48	0.981
		Group An	42	43.52	
	12 Months	Group Am	44	42.97	0.702
		Group An	42	44.06	
	24 Months	Group Am	44	44.43	0.581
		Group An	42	42.52	
Marginal discoloration	Baseline	Group Am	44	43.5	1
		Group An	42	43.5	
	6 Months	Group Am	44	44.43	0.422
		Group An	42	42.52	
	12 Months	Group Am	44	43.92	0.783
		Group An	42	43.06	
	24 Months	Group Am	44	44.9	0.443
		Group An	42	42.04	
Marginal adaptation	Baseline	Group Am	44	43.5	1
		Group An	42	43.5	
	6 Months	Group Am	44	44.93	0.087
		Group An	42	42	
	12 Months	Group Am	44	44.43	0.422
		Group An	42	42.52	
	24 Months	Group Am	44	43.97	0.76
		Group An	42	43.01	
Secondary caries	Baseline	Group Am	44	43.5	1
		Group An	42	43.5	
	6 Months	Group Am	44	43.98	0.329
		Group An	42	43	
	12 Months	Group Am	44	43.97	0.577
		Group An	42	43.01	
	24 Months	Group Am	44	44.43	0.524
		Group An	42	42.52	
Surface texture	Baseline	Group Am	44	43.5	1
		Group An	42	43.5	
	6 Months	Group Am	44	43.45	0.973
		Group An	42	43.55	
	12 Months	Group Am	44	43.43	0.966
		Group An	42	43.57	
	24 Months	Group Am	44	44.43	0.601
		Group An	42	42.52	
Anatomical form	Baseline	Group Am	44	43.5	1
		Group An	42	43.5	
	6 Months	Group Am	44	43.98	0.329
		Group An	42	43	
	12 Months	Group Am	44	44.44	0.326
		Group An	42	42.51	
	24 Months	Group Am	44	44.43	0.482
		Group An	42	42.52	
Post-operative sensitivity	Baseline	Group Am	44	42.98	0.532
		Group An	42	44.05	
	6 Months	Group Am	44	43.98	0.329
		Group An	42	43	
	12 Months	Group Am	44	44.93	0.087
		Group An	42	42	
	24 Months	Group Am	44	43.5	1
		Group An	42	43.5	

*Statistical significance set at 0.05; Group Am-Microhybrid (Agreeableness) Vs Group An-Nanoceramic (Agreeableness).

**Table 5:** Comparison of Group Nm with Group Nn at baseline, 6, 12 and 24 months among different parameters.

		Groups	N	Mean rank	P value
Retention	Baseline	Group Nm	48	48	1
		Group Nn	47	48	
	6 Months	Group Nm	48	48	1
		Group Nn	47	48	
	12 Months	Group Nm	48	48	1
		Group Nn	47	48	
	24 Months	Group Nm	48	46.52	0.243
		Group Nn	47	49.51	
Color match	Baseline	Group Nm	48	48.49	0.322
		Group Nn	47	47.5	
	6 Months	Group Nm	48	48.47	0.665
		Group Nn	47	47.52	
	12 Months	Group Nm	48	47.04	0.552
		Group Nn	47	48.98	
	24 Months	Group Nm	48	45.23	0.199
		Group Nn	47	50.83	
Marginal discoloration	Baseline	Group Nm	48	48	1
		Group Nn	47	48	
	6 Months	Group Nm	48	46.99	0.299
		Group Nn	47	49.03	
	12 Months	Group Nm	48	47.52	0.774
		Group Nn	47	48.49	
	24 Months	Group Nm	48	46.24	0.414
		Group Nn	47	49.8	
Marginal adaptation	Baseline	Group Nm	48	48	1
		Group Nn	47	48	
	6 Months	Group Nm	48	48	1
		Group Nn	47	48	
	12 Months	Group Nm	48	48	1
		Group Nn	47	48	
	24 Months	Group Nm	48	44.98	0.128
		Group Nn	47	51.09	
Secondary caries	Baseline	Group Nm	48	48	1
		Group Nn	47	48	
	6 Months	Group Nm	48	48	1
		Group Nn	47	48	
	12 Months	Group Nm	48	47.52	0.658
		Group Nn	47	48.49	
	24 Months	Group Nm	48	46.48	0.328
		Group Nn	47	49.55	
Surface texture	Baseline	Group Nm	48	48	1
		Group Nn	47	48	
	6 Months	Group Nm	48	46.99	0.299
		Group Nn	47	49.03	
	12 Months	Group Nm	48	47.04	0.578
		Group Nn	47	48.98	
	24 Months	Group Nm	48	45.25	0.223
		Group Nn	47	50.81	
Anatomical form	Baseline	Group Nm	48	48	1
		Group Nn	47	48	
	6 Months	Group Nm	48	48.49	0.322
		Group Nn	47	47.5	
	12 Months	Group Nm	48	48	1
		Group Nn	47	48	
	24 Months	Group Nm	48	46.48	0.328
		Group Nn	47	49.55	
Post-operative sensitivity	Baseline	Group Nm	48	47.99	0.988
		Group Nn	47	48.01	
	6 Months	Group Nm	48	48.49	0.322
		Group Nn	47	47.5	
	12 Months	Group Nm	48	47.52	0.658
		Group Nn	47	48.49	
	24 Months	Group Nm	48	46.48	0.328
		Group Nn	47	49.55	

*Statistical significance set at 0.05; Group Nm-Microhybrid Group (Neuroticism) Vs Group Nn-Nanoceramic (Neuroticism).

**Table 6:** Comparison of Group Am with Group Nm at baseline, 6, 12 and 24 months among different parameters.

		Groups	N	Mean rank	P value
Retention	Baseline	Group Am	44	46.5	1
		Group Nm	48	46.5	
	6 Months	Group Am	44	47.05	0.296
		Group Nm	48	46	
	12 Months	Group Am	44	46.05	0.611
		Group Nm	48	46.92	
	24 Months	Group Am	44	47.14	0.577
		Group Nm	48	45.92	
Color match	Baseline	Group Am	44	46.55	0.951
		Group Nm	48	46.46	
	6 Months	Group Am	44	46.13	0.743
		Group Nm	48	46.84	
	12 Months	Group Am	44	46.15	0.814
		Group Nm	48	46.82	
	24 Months	Group Am	44	46.02	0.812
		Group Nm	48	46.94	
Marginal discoloration	Baseline	Group Am	44	46.5	1
		Group Nm	48	46.5	
	6 Months	Group Am	44	48.19	0.138
		Group Nm	48	44.95	
	12 Months	Group Am	44	46.7	0.904
		Group Nm	48	46.31	
	24 Months	Group Am	44	46.6	0.962
		Group Nm	48	46.41	
Marginal adaptation	Baseline	Group Am	44	46.5	1
		Group Nm	48	46.5	
	6 Months	Group Am	44	48.14	0.067
		Group Nm	48	45	
	12 Months	Group Am	44	46.64	0.924
		Group Nm	48	46.38	
	24 Months	Group Am	44	46.38	0.943
		Group Nm	48	46.61	
Secondary caries	Baseline	Group Am	44	46.5	1
		Group Nm	48	46.5	
	6 Months	Group Am	44	47.05	0.296
		Group Nm	48	46	
	12 Months	Group Am	44	46.57	0.947
		Group Nm	48	46.44	
	24 Months	Group Am	44	47.77	0.418
		Group Nm	48	45.33	
Surface texture	Baseline	Group Am	44	46.5	1
		Group Nm	48	46.5	
	6 Months	Group Am	44	48.19	0.138
		Group Nm	48	44.95	
	12 Months	Group Am	44	46.7	0.904
		Group Nm	48	46.31	
	24 Months	Group Am	44	45.61	0.677
		Group Nm	48	47.31	
Anatomical form	Baseline	Group Am	44	46.5	1
		Group Nm	48	46.5	
	6 Months	Group Am	44	46.56	0.938
		Group Nm	48	46.45	
	12 Months	Group Am	44	46.6	0.934
		Group Nm	48	46.41	
	24 Months	Group Am	44	47.25	0.617
		Group Nm	48	45.81	
Post-operative sensitivity	Baseline	Group Am	44	46.55	0.951
		Group Nm	48	46.46	
	6 Months	Group Am	44	46.56	0.938
		Group Nm	48	46.45	
	12 Months	Group Am	44	47.09	0.605
		Group Nm	48	45.96	
	24 Months	Group Am	44	46.73	0.873
		Group Nm	48	46.29	

*Statistical significance set at 0.05; Group Am-Microhybrid (Agreeableness) Vs Group Nm-Microhybrid Group (Neuroticism).

**Table 7:** Comparison of Group An with Group Nn at baseline, 6, 12 and 24 months among different parameters.

		Groups	N	Mean rank	P value
Retention	Baseline	Group An	42	45	1
		Group Nn	47	45	
	6 Months	Group An	42	45	1
		Group Nn	47	45	
	12 Months	Group An	42	44	0.179
		Group Nn	47	45.89	
	24 Months	Group An	42	43.08	0.128
		Group Nn	47	46.71	
Color match	Baseline	Group An	42	45.56	0.29
		Group Nn	47	44.5	
	6 Months	Group An	42	45.12	0.909
		Group Nn	47	44.89	
	12 Months	Group An	42	44.24	0.657
		Group Nn	47	45.68	
	24 Months	Group An	42	40.70	0.045*
		Group Nn	47	48.84	
Marginal discoloration	Baseline	Group An	42	45	1
		Group Nn	47	45	
	6 Months	Group An	42	44.62	0.742
		Group Nn	47	45.34	
	12 Months	Group An	42	44.24	0.657
		Group Nn	47	45.68	
	24 Months	Group An	42	41.76	0.131
		Group Nn	47	47.89	
Marginal adaptation	Baseline	Group An	42	45	1
		Group Nn	47	45	
	6 Months	Group An	42	45	1
		Group Nn	47	45	
	12 Months	Group An	42	44.1	0.472
		Group Nn	47	45.81	
	24 Months	Group An	42	41.27	0.066
		Group Nn	47	48.33	
Secondary caries	Baseline	Group An	42	45	1
		Group Nn	47	45	
	6 Months	Group An	42	45	1
		Group Nn	47	45	
	12 Months	Group An	42	44.05	0.36
		Group Nn	47	45.85	
	24 Months	Group An	42	43.68	0.425
		Group Nn	47	46.18	
Surface texture	Baseline	Group An	42	45	1
		Group Nn	47	45	
	6 Months	Group An	42	45.74	0.585
		Group Nn	47	44.34	
	12 Months	Group An	42	44.29	0.696
		Group Nn	47	45.64	
	24 Months	Group An	42	40.3	0.036*
		Group Nn	47	49.2	
Anatomical form	Baseline	Group An	42	45	1
		Group Nn	47	45	
	6 Months	Group An	42	45	1
		Group Nn	47	45	
	12 Months	Group An	42	44.05	0.36
		Group Nn	47	45.85	
	24 Months	Group An	42	43.15	0.245
		Group Nn	47	46.65	
Post-operative sensitivity	Baseline	Group An	42	45.62	0.494
		Group Nn	47	44.45	
	6 Months	Group An	42	45	1
		Group Nn	47	45	
	12 Months	Group An	42	43.5	0.098
		Group Nn	47	46.34	
	24 Months	Group An	42	43.68	0.425
		Group Nn	47	46.18	

*Statistical significance set at 0.05; Group An Nanoceramic (Agreeableness) Vs Group Nn Nanoceramic (Neuroticism).

**Table 8:** Comparison of Group A with Group N at baseline, 6, 12 and 24 months among different parameters of combined (Micro-hybrid + Nano-hybrid) restoration performance.

		Group	N	Mean rank	P value
Retention	Baseline	Group A	86	91	1
		Group N	95	91	
	6 Months	Group A	86	91.55	0.293
		Group N	95	90.5	
	12 Months	Group A	86	89.56	0.215
		Group N	95	92.31	
	24 Months	Group A	86	89.76	0.463
		Group N	95	92.13	
Color match	Baseline	Group A	86	91.6	0.504
		Group N	95	90.45	
	6 Months	Group A	86	90.74	0.865
		Group N	95	91.24	
	12 Months	Group A	86	89.88	0.623
		Group N	95	92.01	
	24 Months	Group A	86	86.29	0.108
		Group N	95	95.26	
Marginal discoloration	Baseline	Group A	86	91	1
		Group N	95	91	
	6 Months	Group A	86	92.34	0.409
		Group N	95	89.79	
	12 Months	Group A	86	90.46	0.822
		Group N	95	91.49	
	24 Months	Group A	86	87.9	0.304
		Group N	95	93.81	
Marginal adaptation	Baseline	Group A	86	91	1
		Group N	95	91	
	6 Months	Group A	86	92.66	0.067
		Group N	95	89.5	
	12 Months	Group A	86	90.26	0.695
		Group N	95	91.67	
	24 Months	Group A	86	87.22	0.159
		Group N	95	94.42	
Secondary caries	Baseline	Group A	86	91	1
		Group N	95	91	
	6 Months	Group A	86	91.55	0.293
		Group N	95	90.5	
	12 Months	Group A	86	90.13	0.552
		Group N	95	91.78	
	24 Months	Group A	86	90.99	0.996
		Group N	95	91.01	
Surface texture	Baseline	Group A	86	91	1
		Group N	95	91	
	6 Months	Group A	86	93.44	0.166
		Group N	95	88.79	
	12 Months	Group A	86	90.5	0.841
		Group N	95	91.45	
	24 Months	Group A	86	85.48	0.073
		Group N	95	96	
Anatomical form	Baseline	Group A	86	91	1
		Group N	95	91	
	6 Months	Group A	86	91.06	0.937
		Group N	95	90.95	
	12 Months	Group A	86	90.17	0.61
		Group N	95	91.75	
	24 Months	Group A	86	89.94	0.627
		Group N	95	91.96	
Post-operative sensitivity	Baseline	Group A	86	91.66	0.572
		Group N	95	90.41	
	6 Months	Group A	86	91.06	0.937
		Group N	95	90.95	
	12 Months	Group A	86	90.13	0.552
		Group N	95	91.78	
	24 Months	Group A	86	89.94	0.627
		Group N	95	91.96	

*Statistical significance set at 0.05; Group A (Agreeableness) Vs Group N (Neuroticism).

**Table 9:** Inter Rater-Reliability statistics (Evaluator I VS Evaluator II).

	Baseline Kappa Score	Interpretation	24 months Kappa Score	Interpretation
Retention	NA^a^		1	Almost perfect
Color match	0.854	Almost Perfect	0.856	Almost perfect
Marginal discoloration	NA^a^		0.941	Almost perfect
Marginal adaptation	NA^a^		0.945	Almost perfect
Secondary caries	NA^a^		0.770	Substantial
Surface texture	NA^a^		0.89	Almost perfect
Anatomic form	NA^a^		0.889	Almost perfect
Post operative sensitivity	1	Almost Perfect	0.971	Almost perfect

^a^No statistics are computed because R1 and R2 are constants.

## Discussion

Numerous researchers have established that personality traits have an impact on various health-related outcomes, out of which neuroticism is the most studied personality trait from the health point of view^29^. Studies indicate high prevalence of dental caries and poor oral health are common among neurotic people^30,31^. Among the five personality traits, we selected only two groups (higher neuroticism and higher agreeableness) since they are largely contrasting in nature and experience opposite patterns in disease incidence and progression. Additionally, this study compares the new generation ormocer based Nano-ceramic and universal Micro-hybrid composite in occlusal Class I cavity preparation among patients with different psychological traits.

The results demonstrate that the clinical performance of both Micro-hybrid (Spectrum TPH) and Nano-ceramic (Ceram X mono) composites among same personality groups during the 24-month follow-up period was found to be excellent with no statistically significant difference (Tables 4 and 5), which is similar to previous study, by Schirrmeister et al.^32^. However, all previous studies so far were done in populations without the control of personality trait, patient’s age, cavity type, temporomandibular joint (TMJ) and occlusion statuses unlike the present study, hence the results of this study are more reliable.

The most important factors, which influence the survival of dental restorations, are patient related factors, operator skills, materials and tooth-related factors^33,34^. The primary objective of this study is to find the patient related factor of personality’s influence on the prognosis of restoration while controlling other above-mentioned factors. In a systematic review conducted by Shisei Kubo^35^, no effect of gender, age or cavity size (especially in class I cavity) was observed from many articles that studied the prognosis of composite restoration that is similar to the multi-variate analysis results of the current study (Supplementary Tables 10–25).

Neuroticism group Nano-ceramic restoration had more surface roughness than Agreeableness group Nano-ceramic restoration. Surface roughness is an eventual sequela of wear. Wear can be clinically visualized as surface roughness, loss of the contour, plaque accumulation, surface discoloration, cracks and fracture^36^. According to Turssi et al*.*^37^, cause of restoration wear is mainly due to the occlusion type, chewing characteristics, brushing and parafunctional habits such as bruxism. Among these chewing forces and chewing characteristics play an important role in wear and surface roughness of composite.

Lutz et al.^38^ reported chewing pressure is directly proportional to wear and volume loss of the composite materials. Lambrechts et al.^39^ observed two times greater wear in enamel when the occlusal load was increased to 78 N from 20 N and this wear pattern is similar in composite restorations as well. In a previous study, people with personality traits such as neuroticism are found to have higher bite force compared to those with the agreeableness trait and the mean difference was around 100 Newton and this could be one of the reasons for the increased surface roughness among the neuroticism patients with Nano-ceramic restorations^40^. Tsujimoto et al*.*^41^ stated that excessive occlusal forces might lead to restoration’s surface roughness ultimately losing its shape.

Chewing characteristics also play a significant role in wear and surface roughness. Individuals with higher scores of neuroticism experience hyperactivity of the temporalis muscle, parafunctional habits such as frequent clenching and grinding, binge eating disorder (BED) and also generate increased speed and frequency of chewing strokes^42,43^. All the above conditions can lead to altered chewing pattern among neurotic people which could be an important reason for the observed study results. This was also supported by a previous study finding that stated that chewing pattern is an etiological factor for increased occlusal wear^44^.

Micro-hybrid composites performed better than Nano-ceramic among neuroticism patients. The current study results are contrary to research that demonstrate that larger particle sizes have an unfavorable effect on the wear resistance of dental composites^45^. Although many studies found Nano-hybrid performed better than Micro-hybrid in wear^14,15^, it is also inferred that wear resistance and surface roughness of dental composite resin available in market is more of material dependent rather based on their generic categories usually classified on the basis of filler loading and resin matrix^46^.

Ormocer based Nano-ceramic composite (Ceram X mono), because of its smaller particle size could have easily succumbed to the effects of altered chewing dynamics, increased masticatory forces and consumption of dense abrasive foods^47^ which are highly notable characteristics of higher neuroticism individuals. Ceram X composites contain Nano-fillers, but their wider-diameter glass fillers (mean size 1.2–1.6 µm) can easily rip out leading to surface imperfections^48^. However it should be noted, Nano-ceramic (Ceram X) performance in spite of its material limitations were similar in performance to Micro-hybrid spectrum TPH when compared among agreeableness personality groups (Table 4), clearly reiterating the influence of personality related characteristics in the performance of Nano-ceramic composite rather than material per se.

Neuroticism patient’s Nano-ceramic restoration had more discoloration than Agreeableness patient’s Nano-ceramic restoration. Increased surface roughness of Ceram X mono restorations among higher neuroticism patients led to their accelerated surface discoloration as well. Also individuals with higher neuroticism trait are vulnerable to lead an unhealthy lifestyle and are more prone to adverse habits such as substance abuse (increased amounts of smoking and alcohol addiction)^49^, however these interpretations cannot be generalized but can serve as a predictor on the outcome of results. Acid and alcohol molecules penetrate in to the resin matrix causing softening of the composite surface. This could potentially affect the surface integrity of composite resins leading to its increased discoloration and staining^50^.

Overall higher deterioration and total failure rate of restorations were observed among neuroticism group when compared to agreeableness group. Longer follow up is needed to evaluate the performance of restoration among these group. The complex action of chewing and bite force is influenced by many factors such as age, gender, craniofacial morphology, periodontal support, temporomandibular joint health and dental status^51^. Although best efforts were taken to minimize the effect of these confounding factors by stringent sample selection, nevertheless these confounder’s influence cannot be completely eliminated which is one of the limitations of this study. Clinical performance of composite materials is also influenced by individual patient related unique factors such as dietary habits and oral hygiene which is difficult to be standardized among the participants of this study which is another limitation of this study.

## Conclusion

Assessing patient’s personality before the dental treatment may provide valuable information. As high neurotic trait individuals are often associated with significant dental risk factors, counselling can be given explaining the importance of lifestyle habits and oral hygiene procedures on the treatment prognosis. Clinical Psychologist can be included in the dental team for treating patients to offer psycho-counselling and to intervene on stress management for patients with high neurotic traits to control dental related parafunctional habits. Hence, from a clinical point of view, assessment of personality traits will be useful in recommending a suitable restorative dental material or appropriate prosthesis design, thereby overall improving the quality of treatment provided. However, this is a hypothesis generating study and further studies should be done to test its reliability among different populations.

## Supplementary Information

Supplementary Information.

Supplementary Tables.

## Data Availability

Data is available at reasonable request.

## Abbreviations

BFI: Big five inventory
CONSORT: Consolidated standards of reporting trials
LED: Light emitting diode
TMJ: Temporomandibular joint
USPHS: United States public health service
