# Supplementary material for: Neurotic personality trait as a predictor in the prognosis of composite restorations: A 24-month clinical follow up study
Source: Sci Rep. 2021 Aug 25;11:17179. doi: 10.1038/s41598-021-96229-3 (PMC8387411; doi:10.1038/s41598-021-96229-3)
Supplement: Supplementary file 1 — Supplementary Information. [file 41598_2021_96229_MOESM1_ESM.docx]

**APPENDIX**

**Appendix I: BFI questionnaire**

**How I am in general**

Here are a number of characteristics that may or may not apply to you. For example, do you agree that you are someone who *likes to spend time with others*? Please write a number next to each statement to indicate the extent to which **you agree or disagree with that statement.**

| **1**  Disagree  Strongly | **2**  Disagree  a little | **3**  Neither agree  nor disagree | **4**  Agree  a little | **5**  Agree  strongly |
| --- | --- | --- | --- | --- |

**I am someone who…**

1. _____ Is talkative
2. _____ Tends to find fault with others
3. _____ Does a thorough job
4. _____ Is depressed, blue
5. _____ Is original, comes up with new ideas
6. _____ Is reserved
7. _____ Is helpful and unselfish with others
8. _____ Can be somewhat careless
9. _____ Is relaxed, handles stress well.
10. _____ Is curious about many different things
11. _____ Is full of energy
12. _____ Starts quarrels with others
13. _____ Is a reliable worker
14. _____ Can be tense
15. _____ Is ingenious, a deep thinker
16. _____ Generates a lot of enthusiasm
17. _____ Has a forgiving nature
18. _____ Tends to be disorganized
19. _____ Worries a lot
20. _____ Has an active imagination
21. _____ Tends to be quiet
22. _____ Is generally trusting
23. _____ Tends to be lazy
24. _____ Is emotionally stable, not easily upset
25. _____ Is inventive
26. _____ Has an assertive personality
27. _____ Can be cold and aloof
28. _____ Perseveres until the task is finished
29. _____ Can be moody
30. _____ Values artistic, aesthetic experiences
31. _____ Is sometimes shy, inhibited
32. _____ Is considerate and kind to almost everyone
33. _____ Does things efficiently
34. _____ Remains calm in tense situations
35. _____ Prefers work that is routine
36. _____ Is outgoing, sociable
37. _____ Is sometimes rude to others
38. _____ Makes plans and follows through with them
39. _____ Gets nervous easily
40. _____ Likes to reflect, play with ideas
41. _____ Has few artistic interests
42. _____ Likes to cooperate with others
43. _____ Is easily distracted
44. _____ Is sophisticated in art, music, or literature

**SCORING INSTRUCTIONS**

To score the BFI, you’ll first need to **reverse-score** all negatively-keyed items:

Extraversion: 6, 21, 31

Agreeableness: 2, 12, 27, 37

Conscientiousness: 8, 18, 23, 43

Neuroticism: 9, 24, 34

Openness: 35, 41

To recode these items, you should subtract your score for all reverse-scored items from 6. For example, if you gave yourself a 5, compute 6 minus 5 and your recoded score is 1. That is, a score of 1 becomes 5, 2 becomes 4, 3 remains 3, 4 becomes 2, and 5 becomes 1.

Next, you will create scale scores by ***averaging*** the following items for each B5 domain (where R indicates using the reverse-scored item).

Extraversion: 1, 6R 11, 16, 21R, 26, 31R, 36

Agreeableness: 2R, 7, 12R, 17, 22, 27R, 32, 37R, 42

Conscientiousness: 3, 8R, 13, 18R, 23R, 28, 33, 38, 43R

Neuroticism: 4, 9R, 14, 19, 24R, 29, 34R, 39

Openness: 5, 10, 15, 20, 25, 30, 35R, 40, 41R, 44

**Appendix II: USPHS evaluation form**

**Modified USPHS criteria / Ryge evaluation criteria**

Alfa: restorations that have satisfactory quality and excellent

clinical standard.

Bravo: restorations satisfactory but not ideal (acceptable).

Charlie: restorations that do not have acceptable quality andmust be replaced by preventive reasons.

Delta: restorations with mobility, fractured or lost.

(A alpha, B bravo, C charlie, D delta)

*A = Highest degree of clinical acceptability; B, C and D = progressively lessening degrees of acceptability

**Retention**

Alfa (A) Complete retention of the restoration

Bravo (B) Mobilization of the restoration, still present

Charlie(C) Loss of the restoration

**Color match**

A The restoration matches the adjacent tooth structure in color, shade or translucency

B Mismatch in color, shade or translucency between the restoration and the adjacent tooth

C The mismatch in colour and translucency is outside the acceptable range of

Tooth color and translucency

**Margin discolouration**

A No discolouration anywhere along the margin between therestoration and the adjacent tooth

B Slight discolouration along the margin between the restoration and the adjacent tooth

C The discolouration penetrated along the margin of therestorative material in a pulpal direction

**Margin adaptation**

A No visible evidence of crevice along the margin

B Visible evidence of a crevice along the margin into which the explorer will penetrate

C The dentine or the base is exposed

D The restoration is fractured, mobile or missed

**Surface texture**

A The restoration surface is as smooth as the surrounding enamel

B The restoration surface is rougher than the surrounding enamel

C There is a crevice and fracture on the surface of the restoration

**Anatomical form**

A The restoration is continuous with existing anatomical form

B The restoration is discontinuous with existing anatomical form,but the material is not sufficient to expose dentine or base

C Sufficient material lost to expose dentine or base

**Secondary caries**

A No evidence of caries

B Evidence of caries along the margin of the restoration

**Post-operative sensitivity**

A No post-operative sensitivity at any time during the restorativeprocess and the study period

B Experience of sensitivity at any time during the restorative process and the study period
