# Supplementary material for: Neurotic personality trait as a predictor in the prognosis of composite restorations: A 24-month clinical follow up study
Source: Sci Rep. 2021 Aug 25;11:17179. doi: 10.1038/s41598-021-96229-3 (PMC8387411; doi:10.1038/s41598-021-96229-3)
Supplement: Supplementary file 2 — Supplementary Tables. [file 41598_2021_96229_MOESM2_ESM.docx]

**Regression Analysis done among the four Groups to check the effect of Independent variables (Gender and Cavity size) on the results of Dependent variable (Restoration performance). (Table 10-25)**

**Table 10:** Comparison of Group Am with Group An at baseline, 6, 12 and 24 months among different parameters of **Males**.

|  |  | Groups | N | Mean Rank | P Value |
| --- | --- | --- | --- | --- | --- |
| RETENTION | Baseline | Group Am | 18 | 19.5 | 1 |
|  |  | Group An | 20 | 19.5 |  |
|  | 6 Months | Group Am | 18 | 20.06 | 0.784 |
|  |  | Group An | 20 | 19 |  |
|  | 12 Months | Group Am | 18 | 20.06 | 0.784 |
|  |  | Group An | 20 | 19 |  |
|  | 24 Months | Group Am | 18 | 19.56 | 0.988 |
|  |  | Group An | 20 | 19.45 |  |
| COLOR MATCH | Baseline | Group Am | 18 | 19.56 | 0.988 |
|  |  | Group An | 20 | 19.45 |  |
|  | 6 Months | Group Am | 18 | 19.67 | 0.942 |
|  |  | Group An | 20 | 19.35 |  |
|  | 12 Months | Group Am | 18 | 19.19 | 0.874 |
|  |  | Group An | 20 | 19.78 |  |
|  | 24 Months | Group Am | 18 | 18.67 | 0.675 |
|  |  | Group An | 20 | 20.25 |  |
| MARGINAL DISCOLORATION | Baseline | Group Am | 18 | 19.5 | 1 |
|  |  | Group An | 20 | 19.5 |  |
|  | 6 Months | Group Am | 18 | 20.06 | 0.784 |
|  |  | Group An | 20 | 19 |  |
|  | 12 Months | Group Am | 18 | 20.22 | 0.718 |
|  |  | Group An | 20 | 18.85 |  |
|  | 24 Months | Group Am | 18 | 19.72 | 0.919 |
|  |  | Group An | 20 | 19.3 |  |
| MARGINAL ADAPTATION | Baseline | Group Am | 18 | 19.5 | 1 |
|  |  | Group An | 20 | 19.5 |  |
|  | 6 Months | Group Am | 18 | 20.06 | 0.784 |
|  |  | Group An | 20 | 19 |  |
|  | 12 Months | Group Am | 18 | 20.14 | 0.74 |
|  |  | Group An | 20 | 18.93 |  |
|  | 24 Months | Group Am | 18 | 19.14 | 0.851 |
|  |  | Group An | 20 | 19.83 |  |
| SECONDARY CARIES | Baseline | Group Am | 18 | 19.5 | 1 |
|  |  | Group An | 20 | 19.5 |  |
|  | 6 Months | Group Am | 18 | 20.06 | 0.784 |
|  |  | Group An | 20 | 19 |  |
|  | 12 Months | Group Am | 18 | 19.58 | 0.965 |
|  |  | Group An | 20 | 19.43 |  |
|  | 24 Months | Group Am | 18 | 18.61 | 0.654 |
|  |  | Group An | 20 | 20.3 |  |
| SURFACE TEXTURE | Baseline | Group Am | 18 | 19.5 | 1 |
|  |  | Group An | 20 | 19.5 |  |
|  | 6 Months | Group Am | 18 | 19.19 | 0.874 |
|  |  | Group An | 20 | 19.78 |  |
|  | 12 Months | Group Am | 18 | 19.75 | 0.897 |
|  |  | Group An | 20 | 19.28 |  |
|  | 24 Months | Group Am | 18 | 19.72 | 0.919 |
|  |  | Group An | 20 | 19.3 |  |
| ANATOMICAL FORM | Baseline | Group Am | 18 | 19.5 | 1 |
|  |  | Group An | 20 | 19.5 |  |
|  | 6 Months | Group Am | 18 | 20.06 | 0.784 |
|  |  | Group An | 20 | 19 |  |
|  | 12 Months | Group Am | 18 | 20.14 | 0.74 |
|  |  | Group An | 20 | 18.93 |  |
|  | 24 Months | Group Am | 18 | 19.61 | 0.965 |
|  |  | Group An | 20 | 19.4 |  |
| POST-OPERATIVE SENSITIVITY | Baseline | Group Am | 18 | 18.5 | 0.613 |
|  |  | Group An | 20 | 20.4 |  |
|  | 6 Months | Group Am | 18 | 20.06 | 0.784 |
|  |  | Group An | 20 | 19 |  |
|  | 12 Months | Group Am | 18 | 20.61 | 0.573 |
|  |  | Group An | 20 | 18.5 |  |
|  | 24 Months | Group Am | 18 | 18.67 | 0.675 |
|  |  | Group An | 20 | 20.25 |  |

*Statistical significance set at 0.05

**Table 11 :** Comparison of Group Nm with Group Nn at baseline, 6, 12 and 24 months among different parameters of **Males**.

|  |  | Groups | N | Mean Rank | P Value |
| --- | --- | --- | --- | --- | --- |
| RETENTION | Baseline | Group Nm | 29 | 30.5 | 1 |
|  |  | Group Nn | 31 | 30.5 |  |
|  | 6 Months | Group Nm | 29 | 30.5 | 1 |
|  |  | Group Nn | 31 | 30.5 |  |
|  | 12 Months | Group Nm | 29 | 30.55 | 0.943 |
|  |  | Group Nn | 31 | 30.45 |  |
|  | 24 Months | Group Nm | 29 | 30.05 | 0.611 |
|  |  | Group Nn | 31 | 30.92 |  |
| COLOR MATCH | Baseline | Group Nm | 29 | 31.03 | 0.301 |
|  |  | Group Nn | 31 | 30 |  |
|  | 6 Months | Group Nm | 29 | 31.07 | 0.518 |
|  |  | Group Nn | 31 | 29.97 |  |
|  | 12 Months | Group Nm | 29 | 29.69 | 0.555 |
|  |  | Group Nn | 31 | 31.26 |  |
|  | 24 Months | Group Nm | 29 | 29.33 | 0.535 |
|  |  | Group Nn | 31 | 31.6 |  |
| MARGINAL DISCOLORATION | Baseline | Group Nm | 29 | 30.5 | 1 |
|  |  | Group Nn | 31 | 30.5 |  |
|  | 6 Months | Group Nm | 29 | 30.53 | 0.962 |
|  |  | Group Nn | 31 | 30.47 |  |
|  | 12 Months | Group Nm | 29 | 30.66 | 0.898 |
|  |  | Group Nn | 31 | 30.35 |  |
|  | 24 Months | Group Nm | 29 | 30.17 | 0.858 |
|  |  | Group Nn | 31 | 30.81 |  |
| MARGINAL ADAPTATION | Baseline | Group Nm | 29 | 30.5 | 1 |
|  |  | Group Nn | 31 | 30.5 |  |
|  | 6 Months | Group Nm | 29 | 30.5 | 1 |
|  |  | Group Nn | 31 | 30.5 |  |
|  | 12 Months | Group Nm | 29 | 31.09 | 0.505 |
|  |  | Group Nn | 31 | 29.95 |  |
|  | 24 Months | Group Nm | 29 | 28.74 | 0.305 |
|  |  | Group Nn | 31 | 32.15 |  |
| SECONDARY CARIES | Baseline | Group Nm | 29 | 30.5 | 1 |
|  |  | Group Nn | 31 | 30.5 |  |
|  | 6 Months | Group Nm | 29 | 30.5 | 1 |
|  |  | Group Nn | 31 | 30.5 |  |
|  | 12 Months | Group Nm | 29 | 30.55 | 0.943 |
|  |  | Group Nn | 31 | 30.45 |  |
|  | 24 Months | Group Nm | 29 | 30.12 | 0.77 |
|  |  | Group Nn | 31 | 30.85 |  |
| SURFACE TEXTURE | Baseline | Group Nm | 29 | 30.5 | 1 |
|  |  | Group Nn | 31 | 30.5 |  |
|  | 6 Months | Group Nm | 29 | 29.53 | 0.338 |
|  |  | Group Nn | 31 | 31.4 |  |
|  | 12 Months | Group Nm | 29 | 29.21 | 0.37 |
|  |  | Group Nn | 31 | 31.71 |  |
|  | 24 Months | Group Nm | 29 | 28.74 | 0.372 |
|  |  | Group Nn | 31 | 32.15 |  |
| ANATOMICAL FORM | Baseline | Group Nm | 29 | 30.5 | 1 |
|  |  | Group Nn | 31 | 30.5 |  |
|  | 6 Months | Group Nm | 29 | 31.03 | 0.301 |
|  |  | Group Nn | 31 | 30 |  |
|  | 12 Months | Group Nm | 29 | 30.6 | 0.918 |
|  |  | Group Nn | 31 | 30.4 |  |
|  | 24 Months | Group Nm | 29 | 29.64 | 0.531 |
|  |  | Group Nn | 31 | 31.31 |  |
| POST-OPERATIVE SENSITIVITY | Baseline | Group Nm | 29 | 30.53 | 0.962 |
|  |  | Group Nn | 31 | 30.47 |  |
|  | 6 Months | Group Nm | 29 | 31.03 | 0.301 |
|  |  | Group Nn | 31 | 30 |  |
|  | 12 Months | Group Nm | 29 | 30.07 | 0.624 |
|  |  | Group Nn | 31 | 30.9 |  |
|  | 24 Months | Group Nm | 29 | 30.6 | 0.932 |
|  |  | Group Nn | 31 | 30.4 |  |

*Statistical significance set at 0.05

**Table 12 :** Comparison of Group Am with Group Nm at baseline, 6, 12 and 24 months among different parameters of **Males**.

|  |  | Groups | N | Mean Rank | P Value |
| --- | --- | --- | --- | --- | --- |
| RETENTION | Baseline | Group Am | 18 | 24 | 1 |
|  |  | Group Nm | 29 | 24 |  |
|  | 6 Months | Group Am | 18 | 24.81 | 0.204 |
|  |  | Group Nm | 29 | 23.5 |  |
|  | 12 Months | Group Am | 18 | 24.31 | 0.731 |
|  |  | Group Nm | 29 | 23.81 |  |
|  | 24 Months | Group Am | 18 | 24.31 | 0.731 |
|  |  | Group Nm | 29 | 23.81 |  |
| COLOR MATCH | Baseline | Group Am | 18 | 24.31 | 0.731 |
|  |  | Group Nm | 29 | 23.81 |  |
|  | 6 Months | Group Am | 18 | 24.67 | 0.587 |
|  |  | Group Nm | 29 | 23.59 |  |
|  | 12 Months | Group Am | 18 | 24.14 | 0.919 |
|  |  | Group Nm | 29 | 23.91 |  |
|  | 24 Months | Group Am | 18 | 21.78 | 0.219 |
|  |  | Group Nm | 29 | 25.38 |  |
| MARGINAL DISCOLORATION | Baseline | Group Am | 18 | 24 | 1 |
|  |  | Group Nm | 29 | 24 |  |
|  | 6 Months | Group Am | 18 | 24.33 | 0.707 |
|  |  | Group Nm | 29 | 23.79 |  |
|  | 12 Months | Group Am | 18 | 24.92 | 0.533 |
|  |  | Group Nm | 29 | 23.43 |  |
|  | 24 Months | Group Am | 18 | 23.33 | 0.73 |
|  |  | Group Nm | 29 | 24.41 |  |
| MARGINAL ADAPTATION | Baseline | Group Am | 18 | 24 | 1 |
|  |  | Group Nm | 29 | 24 |  |
|  | 6 Months | Group Am | 18 | 24.81 | 0.204 |
|  |  | Group Nm | 29 | 23.5 |  |
|  | 12 Months | Group Am | 18 | 24.61 | 0.619 |
|  |  | Group Nm | 29 | 23.62 |  |
|  | 24 Months | Group Am | 18 | 23.19 | 0.608 |
|  |  | Group Nm | 29 | 24.5 |  |
| SECONDARY CARIES | Baseline | Group Am | 18 | 24 | 1 |
|  |  | Group Nm | 29 | 24 |  |
|  | 6 Months | Group Am | 18 | 24.81 | 0.204 |
|  |  | Group Nm | 29 | 23.5 |  |
|  | 12 Months | Group Am | 18 | 24.31 | 0.731 |
|  |  | Group Nm | 29 | 23.81 |  |
|  | 24 Months | Group Am | 18 | 23.36 | 0.603 |
|  |  | Group Nm | 29 | 24.4 |  |
| SURFACE TEXTURE | Baseline | Group Am | 18 | 24 | 1 |
|  |  | Group Nm | 29 | 24 |  |
|  | 6 Months | Group Am | 18 | 25.14 | 0.29 |
|  |  | Group Nm | 29 | 23.29 |  |
|  | 12 Months | Group Am | 18 | 24.92 | 0.533 |
|  |  | Group Nm | 29 | 23.43 |  |
|  | 24 Months | Group Am | 18 | 22.86 | 0.565 |
|  |  | Group Nm | 29 | 24.71 |  |
| ANATOMICAL FORM | Baseline | Group Am | 18 | 24 | 1 |
|  |  | Group Nm | 29 | 24 |  |
|  | 6 Months | Group Am | 18 | 24.33 | 0.707 |
|  |  | Group Nm | 29 | 23.79 |  |
|  | 12 Months | Group Am | 18 | 24.61 | 0.619 |
|  |  | Group Nm | 29 | 23.62 |  |
|  | 24 Months | Group Am | 18 | 24.14 | 0.919 |
|  |  | Group Nm | 29 | 23.91 |  |
| POST-OPERATIVE SENSITIVITY | Baseline | Group Am | 18 | 23.5 | 0.431 |
|  |  | Group Nm | 29 | 24.31 |  |
|  | 6 Months | Group Am | 18 | 24.33 | 0.707 |
|  |  | Group Nm | 29 | 23.79 |  |
|  | 12 Months | Group Am | 18 | 25.08 | 0.314 |
|  |  | Group Nm | 29 | 23.33 |  |
|  | 24 Months | Group Am | 18 | 24.14 | 0.919 |
|  |  | Group Nm | 29 | 23.91 |  |

*Statistical significance set at 0.05

**Table 13:** Comparison of Group An with Group Nn at baseline, 6, 12 and 24 months among different parameters of **Males**.

|  |  | Groups | N | Mean Rank | P Value |
| --- | --- | --- | --- | --- | --- |
| RETENTION | Baseline | Group An | 20 | 26 | 1 |
|  |  | Group Nn | 31 | 26 |  |
|  | 6 Months | Group An | 20 | 26 | 1 |
|  |  | Group Nn | 31 | 26 |  |
|  | 12 Months | Group An | 20 | 25.5 | 0.422 |
|  |  | Group Nn | 31 | 26.32 |  |
|  | 24 Months | Group An | 20 | 25.8 | 0.85 |
|  |  | Group Nn | 31 | 26.13 |  |
| COLOR MATCH | Baseline | Group An | 20 | 26.78 | 0.213 |
|  |  | Group Nn | 31 | 25.5 |  |
|  | 6 Months | Group An | 20 | 27.05 | 0.32 |
|  |  | Group Nn | 31 | 25.32 |  |
|  | 12 Months | Group An | 20 | 25.83 | 0.915 |
|  |  | Group Nn | 31 | 26.11 |  |
|  | 24 Months | Group An | 20 | 23.78 | 0.28 |
|  |  | Group Nn | 31 | 27.44 |  |
| MARGINAL DISCOLORATION | Baseline | Group An | 20 | 26 | 1 |
|  |  | Group Nn | 31 | 26 |  |
|  | 6 Months | Group An | 20 | 25.5 | 0.422 |
|  |  | Group Nn | 31 | 26.32 |  |
|  | 12 Months | Group An | 20 | 26.05 | 0.97 |
|  |  | Group Nn | 31 | 25.97 |  |
|  | 24 Months | Group An | 20 | 24.65 | 0.494 |
|  |  | Group Nn | 31 | 26.87 |  |
| MARGINAL ADAPTATION | Baseline | Group An | 20 | 26 | 1 |
|  |  | Group Nn | 31 | 26 |  |
|  | 6 Months | Group An | 20 | 26 | 1 |
|  |  | Group Nn | 31 | 26 |  |
|  | 12 Months | Group An | 20 | 26.28 | 0.752 |
|  |  | Group Nn | 31 | 25.82 |  |
|  | 24 Months | Group An | 20 | 23.98 | 0.29 |
|  |  | Group Nn | 31 | 27.31 |  |
| SECONDARY CARIES | Baseline | Group An | 20 | 26 | 1 |
|  |  | Group Nn | 31 | 26 |  |
|  | 6 Months | Group An | 20 | 26 | 1 |
|  |  | Group Nn | 31 | 26 |  |
|  | 12 Months | Group An | 20 | 26.28 | 0.752 |
|  |  | Group Nn | 31 | 25.82 |  |
|  | 24 Months | Group An | 20 | 26.35 | 0.821 |
|  |  | Group Nn | 31 | 25.77 |  |
| SURFACE TEXTURE | Baseline | Group An | 20 | 26 | 1 |
|  |  | Group Nn | 31 | 26 |  |
|  | 6 Months | Group An | 20 | 26.83 | 0.568 |
|  |  | Group Nn | 31 | 25.47 |  |
|  | 12 Months | Group An | 20 | 25.33 | 0.693 |
|  |  | Group Nn | 31 | 26.44 |  |
|  | 24 Months | Group An | 20 | 22.75 | 0.129 |
|  |  | Group Nn | 31 | 28.1 |  |
| ANATOMICAL FORM | Baseline | Group An | 20 | 26 | 1 |
|  |  | Group Nn | 31 | 26 |  |
|  | 6 Months | Group An | 20 | 26 | 1 |
|  |  | Group Nn | 31 | 26 |  |
|  | 12 Months | Group An | 20 | 25.78 | 0.831 |
|  |  | Group Nn | 31 | 26.15 |  |
|  | 24 Months | Group An | 20 | 25.13 | 0.572 |
|  |  | Group Nn | 31 | 26.56 |  |
| POST-OPERATIVE SENSITIVITY | Baseline | Group An | 20 | 27.05 | 0.32 |
|  |  | Group Nn | 31 | 25.32 |  |
|  | 6 Months | Group An | 20 | 26 | 1 |
|  |  | Group Nn | 31 | 26 |  |
|  | 12 Months | Group An | 20 | 25 | 0.251 |
|  |  | Group Nn | 31 | 26.65 |  |
|  | 24 Months | Group An | 20 | 27.58 | 0.309 |
|  |  | Group Nn | 31 | 24.98 |  |

*Statistical significance set at 0.05

**Multivariate analysis comparison among females between different groups**

**Table 14:** Comparison of Group Am with Group An at baseline, 6, 12 and 24 months among different parameters of **Females.**

|  |  | Groups | N | Mean Rank | P Value |
| --- | --- | --- | --- | --- | --- |
| RETENTION | Baseline | Group Am | 26 | 24.5 | 1 |
|  |  | Group An | 22 | 24.5 |  |
|  | 6 Months | Group Am | 26 | 24.5 | 1 |
|  |  | Group An | 22 | 24.5 |  |
|  | 12 Months | Group Am | 26 | 24.5 | 1 |
|  |  | Group An | 22 | 24.5 |  |
|  | 24 Months | Group Am | 26 | 25.35 | 0.189 |
|  |  | Group An | 22 | 23.5 |  |
| COLOR MATCH | Baseline | Group Am | 26 | 24.5 | 1 |
|  |  | Group An | 22 | 24.5 |  |
|  | 6 Months | Group Am | 26 | 24.5 | 1 |
|  |  | Group An | 22 | 24.5 |  |
|  | 12 Months | Group Am | 26 | 24.35 | 0.863 |
|  |  | Group An | 22 | 24.68 |  |
|  | 24 Months | Group Am | 26 | 26.12 | 0.18 |
|  |  | Group An | 22 | 22.59 |  |
| MARGINAL DISCOLORATION | Baseline | Group Am | 26 | 24.5 | 1 |
|  |  | Group An | 22 | 24.5 |  |
|  | 6 Months | Group Am | 26 | 24.77 | 0.784 |
|  |  | Group An | 22 | 24.18 |  |
|  | 12 Months | Group Am | 26 | 24.27 | 0.828 |
|  |  | Group An | 22 | 24.77 |  |
|  | 24 Months | Group Am | 26 | 25.65 | 0.36 |
|  |  | Group An | 22 | 23.14 |  |
| MARGINAL ADAPTATION | Baseline | Group Am | 26 | 24.5 | 1 |
|  |  | Group An | 22 | 24.5 |  |
|  | 6 Months | Group Am | 26 | 25.35 | 0.189 |
|  |  | Group An | 22 | 23.5 |  |
|  | 12 Months | Group Am | 26 | 24.85 | 0.657 |
|  |  | Group An | 22 | 24.09 |  |
|  | 24 Months | Group Am | 26 | 25.27 | 0.471 |
|  |  | Group An | 22 | 23.59 |  |
| SECONDARY CARIES | Baseline | Group Am | 26 | 24.5 | 1 |
|  |  | Group An | 22 | 24.5 |  |
|  | 6 Months | Group Am | 26 | 24.5 | 1 |
|  |  | Group An | 22 | 24.5 |  |
|  | 12 Months | Group Am | 26 | 24.92 | 0.358 |
|  |  | Group An | 22 | 24 |  |
|  | 24 Months | Group Am | 26 | 26.15 | 0.121 |
|  |  | Group An | 22 | 22.55 |  |
| SURFACE TEXTURE | Baseline | Group Am | 26 | 24.5 | 1 |
|  |  | Group An | 22 | 24.5 |  |
|  | 6 Months | Group Am | 26 | 24.85 | 0.657 |
|  |  | Group An | 22 | 24.09 |  |
|  | 12 Months | Group Am | 26 | 24.27 | 0.828 |
|  |  | Group An | 22 | 24.77 |  |
|  | 24 Months | Group Am | 26 | 25.23 | 0.544 |
|  |  | Group An | 22 | 23.64 |  |
| ANATOMICAL FORM | Baseline | Group Am | 26 | 24.5 | 1 |
|  |  | Group An | 22 | 24.5 |  |
|  | 6 Months | Group Am | 26 | 24.5 | 1 |
|  |  | Group An | 22 | 24.5 |  |
|  | 12 Months | Group Am | 26 | 24.92 | 0.358 |
|  |  | Group An | 22 | 24 |  |
|  | 24 Months | Group Am | 26 | 25.31 | 0.365 |
|  |  | Group An | 22 | 23.55 |  |
| POST-OPERATIVE SENSITIVITY | Baseline | Group Am | 26 | 24.92 | 0.358 |
|  |  | Group An | 22 | 24 |  |
|  | 6 Months | Group Am | 26 | 24.5 | 1 |
|  |  | Group An | 22 | 24.5 |  |
|  | 12 Months | Group Am | 26 | 24.92 | 0.358 |
|  |  | Group An | 22 | 24 |  |
|  | 24 Months | Group Am | 26 | 25.35 | 0.189 |
|  |  | Group An | 22 | 23.5 |  |

*Statistical significance set at 0.05

**Table 15:** Comparison of Group Nm with Group Nn at baseline, 6, 12 and 24 months among different parameters of **Females.**

|  |  | Groups | N | Mean Rank | P Value |
| --- | --- | --- | --- | --- | --- |
| RETENTION | Baseline | Group Nm | 19 | 18 | 1 |
|  |  | Group Nn | 16 | 18 |  |
|  | 6 Months | Group Nm | 19 | 18 | 1 |
|  |  | Group Nn | 16 | 18 |  |
|  | 12 Months | Group Nm | 19 | 17.92 | 0.961 |
|  |  | Group Nn | 16 | 18.09 |  |
|  | 24 Months | Group Nm | 19 | 16.95 | 0.523 |
|  |  | Group Nn | 16 | 19.25 |  |
| COLOR MATCH | Baseline | Group Nm | 19 | 18 | 1 |
|  |  | Group Nn | 16 | 18 |  |
|  | 6 Months | Group Nm | 19 | 17.92 | 0.961 |
|  |  | Group Nn | 16 | 18.09 |  |
|  | 12 Months | Group Nm | 19 | 17.84 | 0.935 |
|  |  | Group Nn | 16 | 18.19 |  |
|  | 24 Months | Group Nm | 19 | 16.79 | 0.461 |
|  |  | Group Nn | 16 | 19.44 |  |
| MARGINAL DISCOLORATION | Baseline | Group Nm | 19 | 18 | 1 |
|  |  | Group Nn | 16 | 18 |  |
|  | 6 Months | Group Nm | 19 | 17 | 0.545 |
|  |  | Group Nn | 16 | 19.19 |  |
|  | 12 Months | Group Nm | 19 | 17.29 | 0.659 |
|  |  | Group Nn | 16 | 18.84 |  |
|  | 24 Months | Group Nm | 19 | 16.74 | 0.441 |
|  |  | Group Nn | 16 | 19.5 |  |
| MARGINAL ADAPTATION | Baseline | Group Nm | 19 | 18 | 1 |
|  |  | Group Nn | 16 | 18 |  |
|  | 6 Months | Group Nm | 19 | 18 | 1 |
|  |  | Group Nn | 16 | 18 |  |
|  | 12 Months | Group Nm | 19 | 17.37 | 0.707 |
|  |  | Group Nn | 16 | 18.75 |  |
|  | 24 Months | Group Nm | 19 | 16.84 | 0.481 |
|  |  | Group Nn | 16 | 19.38 |  |
| SECONDARY CARIES | Baseline | Group Nm | 19 | 18 | 1 |
|  |  | Group Nn | 16 | 18 |  |
|  | 6 Months | Group Nm | 19 | 18 | 1 |
|  |  | Group Nn | 16 | 18 |  |
|  | 12 Months | Group Nm | 19 | 17.45 | 0.731 |
|  |  | Group Nn | 16 | 18.66 |  |
|  | 24 Months | Group Nm | 19 | 16.95 | 0.523 |
|  |  | Group Nn | 16 | 19.25 |  |
| SURFACE TEXTURE | Baseline | Group Nm | 19 | 18 | 1 |
|  |  | Group Nn | 16 | 18 |  |
|  | 6 Months | Group Nm | 19 | 18 | 1 |
|  |  | Group Nn | 16 | 18 |  |
|  | 12 Months | Group Nm | 19 | 18.24 | 0.883 |
|  |  | Group Nn | 16 | 17.72 |  |
|  | 24 Months | Group Nm | 19 | 17.21 | 0.635 |
|  |  | Group Nn | 16 | 18.94 |  |
| ANATOMICAL FORM | Baseline | Group Nm | 19 | 18 | 1 |
|  |  | Group Nn | 16 | 18 |  |
|  | 6 Months | Group Nm | 19 | 18 | 1 |
|  |  | Group Nn | 16 | 18 |  |
|  | 12 Months | Group Nm | 19 | 17.92 | 0.961 |
|  |  | Group Nn | 16 | 18.09 |  |
|  | 24 Months | Group Nm | 19 | 17.42 | 0.731 |
|  |  | Group Nn | 16 | 18.69 |  |
| POST-OPERATIVE SENSITIVITY | Baseline | Group Nm | 19 | 18 | 1 |
|  |  | Group Nn | 16 | 18 |  |
|  | 6 Months | Group Nm | 19 | 18 | 1 |
|  |  | Group Nn | 16 | 18 |  |
|  | 12 Months | Group Nm | 19 | 17.92 | 0.961 |
|  |  | Group Nn | 16 | 18.09 |  |
|  | 24 Months | Group Nm | 19 | 16.47 | 0.35 |
|  |  | Group Nn | 16 | 19.81 |  |

*Statistical significance set at 0.05

**Table 16:** Comparison of Group Am with Group Nm at baseline, 6, 12 and 24 months among different parameters of **Females.**

|  |  | Groups | N | Mean Rank | P Value |
| --- | --- | --- | --- | --- | --- |
| RETENTION | Baseline | Group Am | 26 | 23 | 1 |
|  |  | Group Nm | 19 | 23 |  |
|  | 6 Months | Group Am | 26 | 23 | 1 |
|  |  | Group Nm | 19 | 23 |  |
|  | 12 Months | Group Am | 26 | 22.5 | 0.242 |
|  |  | Group Nm | 19 | 23.68 |  |
|  | 24 Months | Group Am | 26 | 23.23 | 0.75 |
|  |  | Group Nm | 19 | 22.68 |  |
| COLOR MATCH | Baseline | Group Am | 26 | 23 | 1 |
|  |  | Group Nm | 19 | 23 |  |
|  | 6 Months | Group Am | 26 | 22.5 | 0.242 |
|  |  | Group Nm | 19 | 23.68 |  |
|  | 12 Months | Group Am | 26 | 22.69 | 0.709 |
|  |  | Group Nm | 19 | 23.42 |  |
|  | 24 Months | Group Am | 26 | 24.15 | 0.3 |
|  |  | Group Nm | 19 | 21.42 |  |
| MARGINAL DISCOLORATION | Baseline | Group Am | 26 | 23 | 1 |
|  |  | Group Nm | 19 | 23 |  |
|  | 6 Months | Group Am | 26 | 24.1 | 0.13 |
|  |  | Group Nm | 19 | 21.5 |  |
|  | 12 Months | Group Am | 26 | 22.54 | 0.64 |
|  |  | Group Nm | 19 | 23.63 |  |
|  | 24 Months | Group Am | 26 | 23.69 | 0.553 |
|  |  | Group Nm | 19 | 22.05 |  |
| MARGINAL ADAPTATION | Baseline | Group Am | 26 | 23 | 1 |
|  |  | Group Nm | 19 | 23 |  |
|  | 6 Months | Group Am | 26 | 23.73 | 0.221 |
|  |  | Group Nm | 19 | 22 |  |
|  | 12 Months | Group Am | 26 | 22.69 | 0.709 |
|  |  | Group Nm | 19 | 23.42 |  |
|  | 24 Months | Group Am | 26 | 23.46 | 0.64 |
|  |  | Group Nm | 19 | 22.37 |  |
| SECONDARY CARIES | Baseline | Group Am | 26 | 23 | 1 |
|  |  | Group Nm | 19 | 23 |  |
|  | 6 Months | Group Am | 26 | 23 | 1 |
|  |  | Group Nm | 19 | 23 |  |
|  | 12 Months | Group Am | 26 | 22.85 | 0.797 |
|  |  | Group Nm | 19 | 23.21 |  |
|  | 24 Months | Group Am | 26 | 24.27 | 0.199 |
|  |  | Group Nm | 19 | 21.26 |  |
| SURFACE TEXTURE | Baseline | Group Am | 26 | 23 | 1 |
|  |  | Group Nm | 19 | 23 |  |
|  | 6 Months | Group Am | 26 | 23.73 | 0.221 |
|  |  | Group Nm | 19 | 22 |  |
|  | 12 Months | Group Am | 26 | 22.54 | 0.64 |
|  |  | Group Nm | 19 | 23.63 |  |
|  | 24 Months | Group Am | 26 | 23.35 | 0.756 |
|  |  | Group Nm | 19 | 22.53 |  |
| ANATOMICAL FORM | Baseline | Group Am | 26 | 23 | 1 |
|  |  | Group Nm | 19 | 23 |  |
|  | 6 Months | Group Am | 26 | 23 | 1 |
|  |  | Group Nm | 19 | 23 |  |
|  | 12 Months | Group Am | 26 | 22.85 | 0.797 |
|  |  | Group Nm | 19 | 23.21 |  |
|  | 24 Months | Group Am | 26 | 23.58 | 0.485 |
|  |  | Group Nm | 19 | 22.21 |  |
| POST-OPERATIVE SENSITIVITY | Baseline | Group Am | 26 | 23.37 | 0.393 |
|  |  | Group Nm | 19 | 22.5 |  |
|  | 6 Months | Group Am | 26 | 23 | 1 |
|  |  | Group Nm | 19 | 23 |  |
|  | 12 Months | Group Am | 26 | 22.85 | 0.797 |
|  |  | Group Nm | 19 | 23.21 |  |
|  | 24 Months | Group Am | 26 | 23.23 | 0.75 |
|  |  | Group Nm | 19 | 22.68 |  |

*Statistical significance set at 0.05

**Table 17:** Comparison of Group An with Group Nn at baseline, 6, 12 and 24 months among different parameters of **Females.**

|  |  | Groups | N | Mean Rank | P Value |
| --- | --- | --- | --- | --- | --- |
| RETENTION | Baseline | Group An | 22 | 19.5 | 1 |
|  |  | Group Nn | 16 | 19.5 |  |
|  | 6 Months | Group An | 22 | 19.5 | 1 |
|  |  | Group Nn | 16 | 19.5 |  |
|  | 12 Months | Group An | 22 | 19 | 0.759 |
|  |  | Group Nn | 16 | 20.19 |  |
|  | 24 Months | Group An | 22 | 18 | 0.341 |
|  |  | Group Nn | 16 | 21.56 |  |
| COLOR MATCH | Baseline | Group An | 22 | 19.5 | 1 |
|  |  | Group Nn | 16 | 19.5 |  |
|  | 6 Months | Group An | 22 | 19 | 0.759 |
|  |  | Group Nn | 16 | 20.19 |  |
|  | 12 Months | Group An | 22 | 19.18 | 0.849 |
|  |  | Group Nn | 16 | 19.94 |  |
|  | 24 Months | Group An | 22 | 18.05 | 0.356 |
|  |  | Group Nn | 16 | 21.5 |  |
| MARGINAL DISCOLORATION | Baseline | Group An | 22 | 19.5 | 1 |
|  |  | Group Nn | 16 | 19.5 |  |
|  | 6 Months | Group An | 22 | 19.23 | 0.872 |
|  |  | Group Nn | 16 | 19.88 |  |
|  | 12 Months | Group An | 22 | 18.52 | 0.529 |
|  |  | Group Nn | 16 | 20.84 |  |
|  | 24 Months | Group An | 22 | 17.95 | 0.326 |
|  |  | Group Nn | 16 | 21.63 |  |
| MARGINAL ADAPTATION | Baseline | Group An | 22 | 19.5 | 1 |
|  |  | Group Nn | 16 | 19.5 |  |
|  | 6 Months | Group An | 22 | 19.5 | 1 |
|  |  | Group Nn | 16 | 19.5 |  |
|  | 12 Months | Group An | 22 | 18.34 | 0.455 |
|  |  | Group Nn | 16 | 21.09 |  |
|  | 24 Months | Group An | 22 | 18.14 | 0.388 |
|  |  | Group Nn | 16 | 21.38 |  |
| SECONDARY CARIES | Baseline | Group An | 22 | 19.5 | 1 |
|  |  | Group Nn | 16 | 19.5 |  |
|  | 6 Months | Group An | 22 | 19.5 | 1 |
|  |  | Group Nn | 16 | 19.5 |  |
|  | 12 Months | Group An | 22 | 18.5 | 0.529 |
|  |  | Group Nn | 16 | 20.88 |  |
|  | 24 Months | Group An | 22 | 18.32 | 0.455 |
|  |  | Group Nn | 16 | 21.13 |  |
| SURFACE TEXTURE | Baseline | Group An | 22 | 19.5 | 1 |
|  |  | Group Nn | 16 | 19.5 |  |
|  | 6 Months | Group An | 22 | 19.86 | 0.827 |
|  |  | Group Nn | 16 | 19 |  |
|  | 12 Months | Group An | 22 | 19.52 | 0.988 |
|  |  | Group Nn | 16 | 19.47 |  |
|  | 24 Months | Group An | 22 | 18.45 | 0.51 |
|  |  | Group Nn | 16 | 20.94 |  |
| ANATOMICAL FORM | Baseline | Group An | 22 | 19.5 | 1 |
|  |  | Group Nn | 16 | 19.5 |  |
|  | 6 Months | Group An | 22 | 19.5 | 1 |
|  |  | Group Nn | 16 | 19.5 |  |
|  | 12 Months | Group An | 22 | 19 | 0.759 |
|  |  | Group Nn | 16 | 20.19 |  |
|  | 24 Months | Group An | 22 | 18.82 | 0.672 |
|  |  | Group Nn | 16 | 20.44 |  |
| POST-OPERATIVE SENSITIVITY | Baseline | Group An | 22 | 19.5 | 1 |
|  |  | Group Nn | 16 | 19.5 |  |
|  | 6 Months | Group An | 22 | 19.5 | 1 |
|  |  | Group Nn | 16 | 19.5 |  |
|  | 12 Months | Group An | 22 | 19 | 0.759 |
|  |  | Group Nn | 16 | 20.19 |  |
|  | 24 Months | Group An | 22 | 17.5 | 0.201 |
|  |  | Group Nn | 16 | 22.25 |  |

*Statistical significance set at 0.05

**Multivariate analysis comparison among small sized cavity between different groups**

**Table 18:** Comparison of Group Am with Group An at baseline, 6, 12 and 24 months among different parameters of **Small cavity.**

|  |  | Groups | N | Mean Rank | P Value |
| --- | --- | --- | --- | --- | --- |
| RETENTION | Baseline | Group Am | 31 | 31.5 | 1 |
|  |  | Group An | 31 | 31.5 |  |
|  | 6 Months | Group Am | 31 | 32.0 | 0.317 |
|  |  | Group An | 31 | 31.0 |  |
|  | 12 Months | Group Am | 31 | 32.0 | 0.317 |
|  |  | Group An | 31 | 31.0 |  |
|  | 24 Months | Group Am | 31 | 32.0 | 0.557 |
|  |  | Group An | 31 | 31.0 |  |
| COLOR MATCH | Baseline | Group Am | 31 | 32.0 | 0.317 |
|  |  | Group An | 31 | 31.0 |  |
|  | 6 Months | Group Am | 31 | 32.0 | 0.545 |
|  |  | Group An | 31 | 31.0 |  |
|  | 12 Months | Group Am | 31 | 31.6 | 0.967 |
|  |  | Group An | 31 | 31.5 |  |
|  | 24 Months | Group Am | 31 | 32.5 | 0.446 |
|  |  | Group An | 31 | 30.5 |  |
| MARGINAL DISCOLORATION | Baseline | Group Am | 31 | 31.5 | 1 |
|  |  | Group An | 31 | 31.5 |  |
|  | 6 Months | Group Am | 31 | 32.0 | 0.623 |
|  |  | Group An | 31 | 31.0 |  |
|  | 12 Months | Group Am | 31 | 32.1 | 0.663 |
|  |  | Group An | 31 | 31.0 |  |
|  | 24 Months | Group Am | 31 | 32.1 | 0.711 |
|  |  | Group An | 31 | 30.9 |  |
| MARGINAL ADAPTATION | Baseline | Group Am | 31 | 31.5 | 1 |
|  |  | Group An | 31 | 31.5 |  |
|  | 6 Months | Group Am | 31 | 33.0 | 0.078 |
|  |  | Group An | 31 | 30.0 |  |
|  | 12 Months | Group Am | 31 | 32.0 | 0.623 |
|  |  | Group An | 31 | 31.0 |  |
|  | 24 Months | Group Am | 31 | 31.6 | 0.961 |
|  |  | Group An | 31 | 31.4 |  |
| SECONDARY CARIES | Baseline | Group Am | 31 | 31.5 | 1 |
|  |  | Group An | 31 | 31.5 |  |
|  | 6 Months | Group Am | 31 | 32.0 | 0.317 |
|  |  | Group An | 31 | 31.0 |  |
|  | 12 Months | Group Am | 31 | 32.5 | 0.154 |
|  |  | Group An | 31 | 30.5 |  |
|  | 24 Months | Group Am | 31 | 32.5 | 0.395 |
|  |  | Group An | 31 | 30.5 |  |
| SURFACE TEXTURE | Baseline | Group Am | 31 | 31.5 | 1 |
|  |  | Group An | 31 | 31.5 |  |
|  | 6 Months | Group Am | 31 | 31.5 | 0.974 |
|  |  | Group An | 31 | 31.5 |  |
|  | 12 Months | Group Am | 31 | 31.6 | 0.961 |
|  |  | Group An | 31 | 31.4 |  |
|  | 24 Months | Group Am | 31 | 32.5 | 0.481 |
|  |  | Group An | 31 | 30.5 |  |
| ANATOMICAL FORM | Baseline | Group Am | 31 | 31.5 | 1 |
|  |  | Group An | 31 | 31.5 |  |
|  | 6 Months | Group Am | 31 | 32.0 | 0.317 |
|  |  | Group An | 31 | 31.0 |  |
|  | 12 Months | Group Am | 31 | 32.5 | 0.298 |
|  |  | Group An | 31 | 30.5 |  |
|  | 24 Months | Group Am | 31 | 32.5 | 0.395 |
|  |  | Group An | 31 | 30.5 |  |
| POST-OPERATIVE SENSITIVITY | Baseline | Group Am | 31 | 31.0 | 0.557 |
|  |  | Group An | 31 | 32.0 |  |
|  | 6 Months | Group Am | 31 | 32.0 | 0.317 |
|  |  | Group An | 31 | 31.0 |  |
|  | 12 Months | Group Am | 31 | 32.5 | 0.154 |
|  |  | Group An | 31 | 30.5 |  |
|  | 24 Months | Group Am | 31 | 32.0 | 0.633 |
|  |  | Group An | 31 | 31.0 |  |

*Statistical significance set at 0.05

**Table 19:** Comparison of Group Nm with Group Nn at baseline, 6, 12 and 24 months among different parameters of **Small cavity.**

|  |  | Groups | N | Mean Rank | P Value |
| --- | --- | --- | --- | --- | --- |
| RETENTION | Baseline | Group Nm | 31 | 32.0 | 1 |
|  |  | Group Nn | 32 | 32.0 |  |
|  | 6 Months | Group Nm | 31 | 32.0 | 1 |
|  |  | Group Nn | 32 | 32.0 |  |
|  | 12 Months | Group Nm | 31 | 31.5 | 0.539 |
|  |  | Group Nn | 32 | 32.5 |  |
|  | 24 Months | Group Nm | 31 | 30.5 | 0.182 |
|  |  | Group Nn | 32 | 33.4 |  |
| COLOR MATCH | Baseline | Group Nm | 31 | 32.0 | 1 |
|  |  | Group Nn | 32 | 32.0 |  |
|  | 6 Months | Group Nm | 31 | 32.5 | 0.539 |
|  |  | Group Nn | 32 | 31.5 |  |
|  | 12 Months | Group Nm | 31 | 31.6 | 0.734 |
|  |  | Group Nn | 32 | 32.4 |  |
|  | 24 Months | Group Nm | 31 | 30.5 | 0.375 |
|  |  | Group Nn | 32 | 33.5 |  |
| MARGINAL DISCOLORATION | Baseline | Group Nm | 31 | 32.0 | 1 |
|  |  | Group Nn | 32 | 32.0 |  |
|  | 6 Months | Group Nm | 31 | 31.0 | 0.161 |
|  |  | Group Nn | 32 | 33.0 |  |
|  | 12 Months | Group Nm | 31 | 31.6 | 0.734 |
|  |  | Group Nn | 32 | 32.4 |  |
|  | 24 Months | Group Nm | 31 | 30.4 | 0.351 |
|  |  | Group Nn | 32 | 33.6 |  |
| MARGINAL ADAPTATION | Baseline | Group Nm | 31 | 32.0 | 1 |
|  |  | Group Nn | 32 | 32.0 |  |
|  | 6 Months | Group Nm | 31 | 32.0 | 1 |
|  |  | Group Nn | 32 | 32.0 |  |
|  | 12 Months | Group Nm | 31 | 31.6 | 0.681 |
|  |  | Group Nn | 32 | 32.4 |  |
|  | 24 Months | Group Nm | 31 | 29.5 | 0.133 |
|  |  | Group Nn | 32 | 34.4 |  |
| SECONDARY CARIES | Baseline | Group Nm | 31 | 32.0 | 1 |
|  |  | Group Nn | 32 | 32.0 |  |
|  | 6 Months | Group Nm | 31 | 32.0 | 1 |
|  |  | Group Nn | 32 | 32.0 |  |
|  | 12 Months | Group Nm | 31 | 31.5 | 0.589 |
|  |  | Group Nn | 32 | 32.5 |  |
|  | 24 Months | Group Nm | 31 | 30.5 | 0.246 |
|  |  | Group Nn | 32 | 33.4 |  |
| SURFACE TEXTURE | Baseline | Group Nm | 31 | 32.0 | 1 |
|  |  | Group Nn | 32 | 32.0 |  |
|  | 6 Months | Group Nm | 31 | 31.0 | 0.321 |
|  |  | Group Nn | 32 | 33.0 |  |
|  | 12 Months | Group Nm | 31 | 30.6 | 0.325 |
|  |  | Group Nn | 32 | 33.4 |  |
|  | 24 Months | Group Nm | 31 | 29.5 | 0.167 |
|  |  | Group Nn | 32 | 34.4 |  |
| ANATOMICAL FORM | Baseline | Group Nm | 31 | 32.0 | 1 |
|  |  | Group Nn | 32 | 32.0 |  |
|  | 6 Months | Group Nm | 31 | 32.5 | 0.31 |
|  |  | Group Nn | 32 | 31.5 |  |
|  | 12 Months | Group Nm | 31 | 32.0 | 0.974 |
|  |  | Group Nn | 32 | 32.0 |  |
|  | 24 Months | Group Nm | 31 | 30.5 | 0.246 |
|  |  | Group Nn | 32 | 33.4 |  |
| POST-OPERATIVE SENSITIVITY | Baseline | Group Nm | 31 | 32.0 | 0.982 |
|  |  | Group Nn | 32 | 32.0 |  |
|  | 6 Months | Group Nm | 31 | 32.0 | 1 |
|  |  | Group Nn | 32 | 32.0 |  |
|  | 12 Months | Group Nm | 31 | 31.5 | 0.589 |
|  |  | Group Nn | 32 | 32.5 |  |
|  | 24 Months | Group Nm | 31 | 30.5 | 0.246 |
|  |  | Group Nn | 32 | 33.4 |  |

*Statistical significance set at 0.05

**Table 20:** Comparison of Group Am with Group Nm at baseline, 6, 12 and 24 months among different parameters of **Small cavity.**

|  |  | Groups | N | Mean Rank | P Value |
| --- | --- | --- | --- | --- | --- |
| RETENTION | Baseline | Group Am | 31 | 31.5 | 1 |
|  |  | Group Nm | 31 | 31.5 |  |
|  | 6 Months | Group Am | 31 | 32.0 | 0.317 |
|  |  | Group Nm | 31 | 31.0 |  |
|  | 12 Months | Group Am | 31 | 31.5 | 1 |
|  |  | Group Nm | 31 | 31.5 |  |
|  | 24 Months | Group Am | 31 | 32.0 | 0.557 |
|  |  | Group Nm | 31 | 31.0 |  |
| COLOR MATCH | Baseline | Group Am | 31 | 32.0 | 0.317 |
|  |  | Group Nm | 31 | 31.0 |  |
|  | 6 Months | Group Am | 31 | 31.5 | 0.974 |
|  |  | Group Nm | 31 | 31.5 |  |
|  | 12 Months | Group Am | 31 | 31.5 | 1 |
|  |  | Group Nm | 31 | 31.5 |  |
|  | 24 Months | Group Am | 31 | 31.1 | 0.799 |
|  |  | Group Nm | 31 | 31.9 |  |
| MARGINAL DISCOLORATION | Baseline | Group Am | 31 | 31.5 | 1 |
|  |  | Group Nm | 31 | 31.5 |  |
|  | 6 Months | Group Am | 31 | 33.0 | 0.078 |
|  |  | Group Nm | 31 | 30.0 |  |
|  | 12 Months | Group Am | 31 | 32.0 | 0.701 |
|  |  | Group Nm | 31 | 31.0 |  |
|  | 24 Months | Group Am | 31 | 31.6 | 0.951 |
|  |  | Group Nm | 31 | 31.4 |  |
| MARGINAL ADAPTATION | Baseline | Group Am | 31 | 31.5 | 1 |
|  |  | Group Nm | 31 | 31.5 |  |
|  | 6 Months | Group Am | 31 | 33.0 | 0.078 |
|  |  | Group Nm | 31 | 30.0 |  |
|  | 12 Months | Group Am | 31 | 32.0 | 0.655 |
|  |  | Group Nm | 31 | 31.0 |  |
|  | 24 Months | Group Am | 31 | 31.6 | 0.961 |
|  |  | Group Nm | 31 | 31.4 |  |
| SECONDARY CARIES | Baseline | Group Am | 31 | 31.5 | 1 |
|  |  | Group Nm | 31 | 31.5 |  |
|  | 6 Months | Group Am | 31 | 32.0 | 0.317 |
|  |  | Group Nm | 31 | 31.0 |  |
|  | 12 Months | Group Am | 31 | 32.0 | 0.57 |
|  |  | Group Nm | 31 | 31.0 |  |
|  | 24 Months | Group Am | 31 | 32.5 | 0.395 |
|  |  | Group Nm | 31 | 30.5 |  |
| SURFACE TEXTURE | Baseline | Group Am | 31 | 31.5 | 1 |
|  |  | Group Nm | 31 | 31.5 |  |
|  | 6 Months | Group Am | 31 | 32.0 | 0.545 |
|  |  | Group Nm | 31 | 31.0 |  |
|  | 12 Months | Group Am | 31 | 32.0 | 0.701 |
|  |  | Group Nm | 31 | 31.0 |  |
|  | 24 Months | Group Am | 31 | 31.6 | 0.951 |
|  |  | Group Nm | 31 | 31.4 |  |
| ANATOMICAL FORM | Baseline | Group Am | 31 | 31.5 | 1 |
|  |  | Group Nm | 31 | 31.5 |  |
|  | 6 Months | Group Am | 31 | 31.5 | 0.982 |
|  |  | Group Nm | 31 | 31.5 |  |
|  | 12 Months | Group Am | 31 | 32.0 | 0.655 |
|  |  | Group Nm | 31 | 31.0 |  |
|  | 24 Months | Group Am | 31 | 32.5 | 0.395 |
|  |  | Group Nm | 31 | 30.5 |  |
| POST-OPERATIVE SENSITIVITY | Baseline | Group Am | 31 | 31.5 | 1 |
|  |  | Group Nm | 31 | 31.5 |  |
|  | 6 Months | Group Am | 31 | 32.0 | 0.317 |
|  |  | Group Nm | 31 | 31.0 |  |
|  | 12 Months | Group Am | 31 | 32.0 | 0.57 |
|  |  | Group Nm | 31 | 31.0 |  |
|  | 24 Months | Group Am | 31 | 32.0 | 0.633 |
|  |  | Group Nm | 31 | 31.0 |  |

*Statistical significance set at 0.05

**Table 21:** Comparison of Group An with Group Nn at baseline, 6, 12 and 24 months among different parameters of **Small cavity.**

|  |  | Groups | N | Mean Rank | P Value |
| --- | --- | --- | --- | --- | --- |
| RETENTION | Baseline | Group An | 31 | 32.0 | 1 |
|  |  | Group Nn | 32 | 32.0 |  |
|  | 6 Months | Group An | 31 | 32.0 | 1 |
|  |  | Group Nn | 32 | 32.0 |  |
|  | 12 Months | Group An | 31 | 31.0 | 0.161 |
|  |  | Group Nn | 32 | 33.0 |  |
|  | 24 Months | Group An | 31 | 30.5 | 0.182 |
|  |  | Group Nn | 32 | 33.4 |  |
| COLOR MATCH | Baseline | Group An | 31 | 32.0 | 1 |
|  |  | Group Nn | 32 | 32.0 |  |
|  | 6 Months | Group An | 31 | 32.0 | 0.982 |
|  |  | Group Nn | 32 | 32.0 |  |
|  | 12 Months | Group An | 31 | 31.5 | 0.696 |
|  |  | Group Nn | 32 | 32.5 |  |
|  | 24 Months | Group An | 31 | 29.1 | 0.066 |
|  |  | Group Nn | 32 | 34.9 |  |
| MARGINAL DISCOLORATION | Baseline | Group An | 31 | 32.0 | 1 |
|  |  | Group Nn | 32 | 32.0 |  |
|  | 6 Months | Group An | 31 | 32.0 | 0.974 |
|  |  | Group Nn | 32 | 32.0 |  |
|  | 12 Months | Group An | 31 | 31.5 | 0.696 |
|  |  | Group Nn | 32 | 32.5 |  |
|  | 24 Months | Group An | 31 | 29.9 | 0.222 |
|  |  | Group Nn | 32 | 34.0 |  |
| MARGINAL ADAPTATION | Baseline | Group An | 31 | 32.0 | 1 |
|  |  | Group Nn | 32 | 32.0 |  |
|  | 6 Months | Group An | 31 | 32.0 | 1 |
|  |  | Group Nn | 32 | 32.0 |  |
|  | 12 Months | Group An | 31 | 31.5 | 0.649 |
|  |  | Group Nn | 32 | 32.5 |  |
|  | 24 Months | Group An | 31 | 29.5 | 0.133 |
|  |  | Group Nn | 32 | 34.4 |  |
| SECONDARY CARIES | Baseline | Group An | 31 | 32.0 | 1 |
|  |  | Group Nn | 32 | 32.0 |  |
|  | 6 Months | Group An | 31 | 32.0 | 1 |
|  |  | Group Nn | 32 | 32.0 |  |
|  | 12 Months | Group An | 31 | 31.0 | 0.161 |
|  |  | Group Nn | 32 | 33.0 |  |
|  | 24 Months | Group An | 31 | 30.5 | 0.246 |
|  |  | Group Nn | 32 | 33.4 |  |
| SURFACE TEXTURE | Baseline | Group An | 31 | 32.0 | 1 |
|  |  | Group Nn | 32 | 32.0 |  |
|  | 6 Months | Group An | 31 | 31.5 | 0.67 |
|  |  | Group Nn | 32 | 32.5 |  |
|  | 12 Months | Group An | 31 | 31.0 | 0.501 |
|  |  | Group Nn | 32 | 33.0 |  |
|  | 24 Months | Group An | 31 | 28.6 | **0.048*** |
|  |  | Group Nn | 32 | 35.3 |  |
| ANATOMICAL FORM | Baseline | Group An | 31 | 32.0 | 1 |
|  |  | Group Nn | 32 | 32.0 |  |
|  | 6 Months | Group An | 31 | 32.0 | 1 |
|  |  | Group Nn | 32 | 32.0 |  |
|  | 12 Months | Group An | 31 | 31.5 | 0.564 |
|  |  | Group Nn | 32 | 32.5 |  |
|  | 24 Months | Group An | 31 | 30.5 | 0.246 |
|  |  | Group Nn | 32 | 33.4 |  |
| POST-OPERATIVE SENSITIVITY | Baseline | Group An | 31 | 32.5 | 0.539 |
|  |  | Group Nn | 32 | 31.5 |  |
|  | 6 Months | Group An | 31 | 32.0 | 1 |
|  |  | Group Nn | 32 | 32.0 |  |
|  | 12 Months | Group An | 31 | 31.0 | 0.161 |
|  |  | Group Nn | 32 | 33.0 |  |
|  | 24 Months | Group An | 31 | 30.5 | 0.246 |
|  |  | Group Nn | 32 | 33.4 |  |

*Statistical significance set at 0.05

**Multivariate analysis comparison among moderate sized cavity between different groups**

**Table 22:** Comparison of Group Am with Group An at baseline, 6, 12 and 24 months among different parameters of **Medium cavity.**

|  |  | Groups | N | Mean Rank | P Value |
| --- | --- | --- | --- | --- | --- |
| RETENTION | Baseline | Group Am | 13 | 12.5 | 1 |
|  |  | Group An | 11 | 12.5 |  |
|  | 6 Months | Group Am | 13 | 12.5 | 1 |
|  |  | Group An | 11 | 12.5 |  |
|  | 12 Months | Group Am | 13 | 12.5 | 1 |
|  |  | Group An | 11 | 12.5 |  |
|  | 24 Months | Group Am | 13 | 12.9 | 0.358 |
|  |  | Group An | 11 | 12.0 |  |
| COLOR MATCH | Baseline | Group Am | 13 | 12.0 | 0.277 |
|  |  | Group An | 11 | 13.1 |  |
|  | 6 Months | Group Am | 13 | 12.0 | 0.277 |
|  |  | Group An | 11 | 13.1 |  |
|  | 12 Months | Group Am | 13 | 11.9 | 0.449 |
|  |  | Group An | 11 | 13.2 |  |
|  | 24 Months | Group Am | 13 | 12.4 | 0.908 |
|  |  | Group An | 11 | 12.6 |  |
| MARGINAL DISCOLORATION | Baseline | Group Am | 13 | 12.5 | 1 |
|  |  | Group An | 11 | 12.5 |  |
|  | 6 Months | Group Am | 13 | 12.9 | 0.358 |
|  |  | Group An | 11 | 12.0 |  |
|  | 12 Months | Group Am | 13 | 12.4 | 0.858 |
|  |  | Group An | 11 | 12.7 |  |
|  | 24 Months | Group Am | 13 | 13.3 | 0.443 |
|  |  | Group An | 11 | 11.6 |  |
| MARGINAL ADAPTATION | Baseline | Group Am | 13 | 12.5 | 1 |
|  |  | Group An | 11 | 12.5 |  |
|  | 6 Months | Group Am | 13 | 12.5 | 1 |
|  |  | Group An | 11 | 12.5 |  |
|  | 12 Months | Group Am | 13 | 12.9 | 0.358 |
|  |  | Group An | 11 | 12.0 |  |
|  | 24 Months | Group Am | 13 | 12.9 | 0.614 |
|  |  | Group An | 11 | 12.1 |  |
| SECONDARY CARIES | Baseline | Group Am | 13 | 12.5 | 1 |
|  |  | Group An | 11 | 12.5 |  |
|  | 6 Months | Group Am | 13 | 12.5 | 1 |
|  |  | Group An | 11 | 12.5 |  |
|  | 12 Months | Group Am | 13 | 12.0 | 0.277 |
|  |  | Group An | 11 | 13.1 |  |
|  | 24 Months | Group Am | 13 | 12.4 | 0.929 |
|  |  | Group An | 11 | 12.6 |  |
| SURFACE TEXTURE | Baseline | Group Am | 13 | 12.5 | 1 |
|  |  | Group An | 11 | 12.5 |  |
|  | 6 Months | Group Am | 13 | 12.4 | 0.858 |
|  |  | Group An | 11 | 12.7 |  |
|  | 12 Months | Group Am | 13 | 12.4 | 0.858 |
|  |  | Group An | 11 | 12.7 |  |
|  | 24 Months | Group Am | 13 | 12.4 | 0.908 |
|  |  | Group An | 11 | 12.6 |  |
| ANATOMICAL FORM | Baseline | Group Am | 13 | 12.5 | 1 |
|  |  | Group An | 11 | 12.5 |  |
|  | 6 Months | Group Am | 13 | 12.5 | 1 |
|  |  | Group An | 11 | 12.5 |  |
|  | 12 Months | Group Am | 13 | 12.5 | 1 |
|  |  | Group An | 11 | 12.5 |  |
|  | 24 Months | Group Am | 13 | 12.5 | 0.952 |
|  |  | Group An | 11 | 12.6 |  |
| POST-OPERATIVE SENSITIVITY | Baseline | Group Am | 13 | 12.5 | 1 |
|  |  | Group An | 11 | 12.5 |  |
|  | 6 Months | Group Am | 13 | 12.5 | 1 |
|  |  | Group An | 11 | 12.5 |  |
|  | 12 Months | Group Am | 13 | 12.9 | 0.358 |
|  |  | Group An | 11 | 12.0 |  |
|  | 24 Months | Group Am | 13 | 12.0 | 0.512 |
|  |  | Group An | 11 | 13.1 |  |

*Statistical significance set at 0.05

**Table 23:** Comparison of Group Nm with Group Nn at baseline, 6, 12 and 24 months among different parameters of **Medium cavity.**

|  |  | Groups | N | Mean Rank | P Value |
| --- | --- | --- | --- | --- | --- |
| RETENTION | Baseline | Group Nm | 17 | 16.5 | 1 |
|  |  | Group Nn | 15 | 16.5 |  |
|  | 6 Months | Group Nm | 17 | 16.5 | 1 |
|  |  | Group Nn | 15 | 16.5 |  |
|  | 12 Months | Group Nm | 17 | 16.9 | 0.348 |
|  |  | Group Nn | 15 | 16.0 |  |
|  | 24 Months | Group Nm | 17 | 16.5 | 0.964 |
|  |  | Group Nn | 15 | 16.5 |  |
| COLOR MATCH | Baseline | Group Nm | 17 | 16.9 | 0.348 |
|  |  | Group Nn | 15 | 16.0 |  |
|  | 6 Months | Group Nm | 17 | 16.4 | 0.928 |
|  |  | Group Nn | 15 | 16.6 |  |
|  | 12 Months | Group Nm | 17 | 16.0 | 0.59 |
|  |  | Group Nn | 15 | 17.1 |  |
|  | 24 Months | Group Nm | 17 | 15.3 | 0.331 |
|  |  | Group Nn | 15 | 17.9 |  |
| MARGINAL DISCOLORATION | Baseline | Group Nm | 17 | 16.5 | 1 |
|  |  | Group Nn | 15 | 16.5 |  |
|  | 6 Months | Group Nm | 17 | 16.4 | 0.928 |
|  |  | Group Nn | 15 | 16.6 |  |
|  | 12 Months | Group Nm | 17 | 16.4 | 0.933 |
|  |  | Group Nn | 15 | 16.6 |  |
|  | 24 Months | Group Nm | 17 | 16.4 | 0.907 |
|  |  | Group Nn | 15 | 16.7 |  |
| MARGINAL ADAPTATION | Baseline | Group Nm | 17 | 16.5 | 1 |
|  |  | Group Nn | 15 | 16.5 |  |
|  | 6 Months | Group Nm | 17 | 16.5 | 1 |
|  |  | Group Nn | 15 | 16.5 |  |
|  | 12 Months | Group Nm | 17 | 16.9 | 0.601 |
|  |  | Group Nn | 15 | 16.0 |  |
|  | 24 Months | Group Nm | 17 | 15.9 | 0.618 |
|  |  | Group Nn | 15 | 17.1 |  |
| SECONDARY CARIES | Baseline | Group Nm | 17 | 16.5 | 1 |
|  |  | Group Nn | 15 | 16.5 |  |
|  | 6 Months | Group Nm | 17 | 16.5 | 1 |
|  |  | Group Nn | 15 | 16.5 |  |
|  | 12 Months | Group Nm | 17 | 16.5 | 0.964 |
|  |  | Group Nn | 15 | 16.5 |  |
|  | 24 Months | Group Nm | 17 | 16.4 | 0.948 |
|  |  | Group Nn | 15 | 16.6 |  |
| SURFACE TEXTURE | Baseline | Group Nm | 17 | 16.5 | 1 |
|  |  | Group Nn | 15 | 16.5 |  |
|  | 6 Months | Group Nm | 17 | 16.5 | 1 |
|  |  | Group Nn | 15 | 16.5 |  |
|  | 12 Months | Group Nm | 17 | 16.9 | 0.697 |
|  |  | Group Nn | 15 | 16.1 |  |
|  | 24 Months | Group Nm | 17 | 16.2 | 0.807 |
|  |  | Group Nn | 15 | 16.9 |  |
| ANATOMICAL FORM | Baseline | Group Nm | 17 | 16.5 | 1 |
|  |  | Group Nn | 15 | 16.5 |  |
|  | 6 Months | Group Nm | 17 | 16.5 | 1 |
|  |  | Group Nn | 15 | 16.5 |  |
|  | 12 Months | Group Nm | 17 | 16.5 | 0.964 |
|  |  | Group Nn | 15 | 16.5 |  |
|  | 24 Months | Group Nm | 17 | 16.4 | 0.948 |
|  |  | Group Nn | 15 | 16.6 |  |
| POST-OPERATIVE SENSITIVITY | Baseline | Group Nm | 17 | 16.5 | 1 |
|  |  | Group Nn | 15 | 16.5 |  |
|  | 6 Months | Group Nm | 17 | 16.9 | 0.348 |
|  |  | Group Nn | 15 | 16.0 |  |
|  | 12 Months | Group Nm | 17 | 16.5 | 0.964 |
|  |  | Group Nn | 15 | 16.5 |  |
|  | 24 Months | Group Nm | 17 | 16.4 | 0.948 |
|  |  | Group Nn | 15 | 16.6 |  |

*Statistical significance set at 0.05

**Table 24:** Comparison of Group Am with Group Nm at baseline, 6, 12 and 24 months among different parameters of **Medium cavity.**

|  |  | Groups | N | Mean Rank | P Value |
| --- | --- | --- | --- | --- | --- |
| RETENTION | Baseline | Group Am | 13 | 15.5 | 1 |
|  |  | Group Nm | 17 | 15.5 |  |
|  | 6 Months | Group Am | 13 | 15.5 | 1 |
|  |  | Group Nm | 17 | 15.5 |  |
|  | 12 Months | Group Am | 13 | 15.0 | 0.382 |
|  |  | Group Nm | 17 | 15.9 |  |
|  | 24 Months | Group Am | 13 | 15.7 | 0.846 |
|  |  | Group Nm | 17 | 15.4 |  |
| COLOR MATCH | Baseline | Group Am | 13 | 15.0 | 0.382 |
|  |  | Group Nm | 17 | 15.9 |  |
|  | 6 Months | Group Am | 13 | 15.0 | 0.382 |
|  |  | Group Nm | 17 | 15.9 |  |
|  | 12 Months | Group Am | 13 | 15.1 | 0.688 |
|  |  | Group Nm | 17 | 15.8 |  |
|  | 24 Months | Group Am | 13 | 15.5 | 1 |
|  |  | Group Nm | 17 | 15.5 |  |
| MARGINAL DISCOLORATION | Baseline | Group Am | 13 | 15.5 | 1 |
|  |  | Group Nm | 17 | 15.5 |  |
|  | 6 Months | Group Am | 13 | 15.7 | 0.846 |
|  |  | Group Nm | 17 | 15.4 |  |
|  | 12 Months | Group Am | 13 | 15.2 | 0.821 |
|  |  | Group Nm | 17 | 15.7 |  |
|  | 24 Months | Group Am | 13 | 15.7 | 0.917 |
|  |  | Group Nm | 17 | 15.4 |  |
| MARGINAL ADAPTATION | Baseline | Group Am | 13 | 15.5 | 1 |
|  |  | Group Nm | 17 | 15.5 |  |
|  | 6 Months | Group Am | 13 | 15.5 | 1 |
|  |  | Group Nm | 17 | 15.5 |  |
|  | 12 Months | Group Am | 13 | 15.1 | 0.688 |
|  |  | Group Nm | 17 | 15.8 |  |
|  | 24 Months | Group Am | 13 | 15.4 | 0.897 |
|  |  | Group Nm | 17 | 15.6 |  |
| SECONDARY CARIES | Baseline | Group Am | 13 | 15.5 | 1 |
|  |  | Group Nm | 17 | 15.5 |  |
|  | 6 Months | Group Am | 13 | 15.5 | 1 |
|  |  | Group Nm | 17 | 15.5 |  |
|  | 12 Months | Group Am | 13 | 15.0 | 0.382 |
|  |  | Group Nm | 17 | 15.9 |  |
|  | 24 Months | Group Am | 13 | 15.8 | 0.777 |
|  |  | Group Nm | 17 | 15.3 |  |
| SURFACE TEXTURE | Baseline | Group Am | 13 | 15.5 | 1 |
|  |  | Group Nm | 17 | 15.5 |  |
|  | 6 Months | Group Am | 13 | 16.8 | 0.1 |
|  |  | Group Nm | 17 | 14.5 |  |
|  | 12 Months | Group Am | 13 | 15.2 | 0.821 |
|  |  | Group Nm | 17 | 15.7 |  |
|  | 24 Months | Group Am | 13 | 14.6 | 0.532 |
|  |  | Group Nm | 17 | 16.2 |  |
| ANATOMICAL FORM | Baseline | Group Am | 13 | 15.5 | 1 |
|  |  | Group Nm | 17 | 15.5 |  |
|  | 6 Months | Group Am | 13 | 15.5 | 1 |
|  |  | Group Nm | 17 | 15.5 |  |
|  | 12 Months | Group Am | 13 | 15.0 | 0.382 |
|  |  | Group Nm | 17 | 15.9 |  |
|  | 24 Months | Group Am | 13 | 15.2 | 0.748 |
|  |  | Group Nm | 17 | 15.7 |  |
| POST-OPERATIVE SENSITIVITY | Baseline | Group Am | 13 | 15.5 | 1 |
|  |  | Group Nm | 17 | 15.5 |  |
|  | 6 Months | Group Am | 13 | 15.0 | 0.382 |
|  |  | Group Nm | 17 | 15.9 |  |
|  | 12 Months | Group Am | 13 | 15.6 | 0.885 |
|  |  | Group Nm | 17 | 15.4 |  |
|  | 24 Months | Group Am | 13 | 15.2 | 0.748 |
|  |  | Group Nm | 17 | 15.7 |  |

*Statistical significance set at 0.05

**Table 25:** Comparison of Group An with Group Nn at baseline, 6, 12 and 24 months among different parameters of **Medium cavity.**

|  |  | Groups | N | Mean Rank | P Value |
| --- | --- | --- | --- | --- | --- |
| RETENTION | Baseline | Group An | 11 | 13.5 | 1 |
|  |  | Group Nn | 15 | 13.5 |  |
|  | 6 Months | Group An | 11 | 13.5 | 1 |
|  |  | Group Nn | 15 | 13.5 |  |
|  | 12 Months | Group An | 11 | 13.5 | 1 |
|  |  | Group Nn | 15 | 13.5 |  |
|  | 24 Months | Group An | 11 | 13.0 | 0.392 |
|  |  | Group Nn | 15 | 13.9 |  |
| COLOR MATCH | Baseline | Group An | 11 | 14.2 | 0.243 |
|  |  | Group Nn | 15 | 13.0 |  |
|  | 6 Months | Group An | 11 | 13.7 | 0.822 |
|  |  | Group Nn | 15 | 13.4 |  |
|  | 12 Months | Group An | 11 | 13.4 | 0.909 |
|  |  | Group Nn | 15 | 13.6 |  |
|  | 24 Months | Group An | 11 | 12.3 | 0.403 |
|  |  | Group Nn | 15 | 14.4 |  |
| MARGINAL DISCOLORATION | Baseline | Group An | 11 | 13.5 | 1 |
|  |  | Group Nn | 15 | 13.5 |  |
|  | 6 Months | Group An | 11 | 13.0 | 0.392 |
|  |  | Group Nn | 15 | 13.9 |  |
|  | 12 Months | Group An | 11 | 13.4 | 0.909 |
|  |  | Group Nn | 15 | 13.6 |  |
|  | 24 Months | Group An | 11 | 12.4 | 0.399 |
|  |  | Group Nn | 15 | 14.3 |  |
| MARGINAL ADAPTATION | Baseline | Group An | 11 | 13.5 | 1 |
|  |  | Group Nn | 15 | 13.5 |  |
|  | 6 Months | Group An | 11 | 13.5 | 1 |
|  |  | Group Nn | 15 | 13.5 |  |
|  | 12 Months | Group An | 11 | 13.0 | 0.392 |
|  |  | Group Nn | 15 | 13.9 |  |
|  | 24 Months | Group An | 11 | 12.2 | 0.271 |
|  |  | Group Nn | 15 | 14.5 |  |
| SECONDARY CARIES | Baseline | Group An | 11 | 13.5 | 1 |
|  |  | Group Nn | 15 | 13.5 |  |
|  | 6 Months | Group An | 11 | 13.5 | 1 |
|  |  | Group Nn | 15 | 13.5 |  |
|  | 12 Months | Group An | 11 | 13.7 | 0.822 |
|  |  | Group Nn | 15 | 13.4 |  |
|  | 24 Months | Group An | 11 | 13.9 | 0.74 |
|  |  | Group Nn | 15 | 13.2 |  |
| SURFACE TEXTURE | Baseline | Group An | 11 | 13.5 | 1 |
|  |  | Group Nn | 15 | 13.5 |  |
|  | 6 Months | Group An | 11 | 14.9 | 0.092 |
|  |  | Group Nn | 15 | 12.5 |  |
|  | 12 Months | Group An | 11 | 13.9 | 0.74 |
|  |  | Group Nn | 15 | 13.2 |  |
|  | 24 Months | Group An | 11 | 12.4 | 0.454 |
|  |  | Group Nn | 15 | 14.3 |  |
| ANATOMICAL FORM | Baseline | Group An | 11 | 13.5 | 1 |
|  |  | Group Nn | 15 | 13.5 |  |
|  | 6 Months | Group An | 11 | 13.5 | 1 |
|  |  | Group Nn | 15 | 13.5 |  |
|  | 12 Months | Group An | 11 | 13.0 | 0.392 |
|  |  | Group Nn | 15 | 13.9 |  |
|  | 24 Months | Group An | 11 | 13.2 | 0.743 |
|  |  | Group Nn | 15 | 13.7 |  |
| POST-OPERATIVE SENSITIVITY | Baseline | Group An | 11 | 13.5 | 1 |
|  |  | Group Nn | 15 | 13.5 |  |
|  | 6 Months | Group An | 11 | 13.5 | 1 |
|  |  | Group Nn | 15 | 13.5 |  |
|  | 12 Months | Group An | 11 | 13.0 | 0.392 |
|  |  | Group Nn | 15 | 13.9 |  |
|  | 24 Months | Group An | 11 | 13.9 | 0.74 |
|  |  | Group Nn | 15 | 13.2 |  |

*Statistical significance set at 0.05
